# Physical activity and acute exercise benefit influenza vaccination response: A systematic review with individual participant data meta-analysis

**DOI:** 10.1371/journal.pone.0268625

**Published:** 2022-06-15

**Authors:** Erika Bohn-Goldbaum, Katherine B. Owen, Vivian Y. J. Lee, Robert Booy, Kate M. Edwards

**Affiliations:** 1 School of Health Sciences, Faculty of Medicine and Health, The University of Sydney, Camperdown, New South Wales, Australia; 2 Charles Perkins Centre, The University of Sydney, Camperdown, New South Wales, Australia; 3 School of Public Health, Faculty of Medicine and Health, The University of Sydney, Camperdown, New South Wales, Australia; 4 School of Sport, Exercise & Rehabilitation Sciences, College of Life & Environmental Sciences, University of Birmingham, Edgbaston, Birmingham, England; 5 The Children’s Hospital at Westmead, Sydney Medical School, The University of Sydney, Westmead, New South Wales, Australia; Prince Sattam Bin Abdulaziz University, College of Applied Medical Sciences, SAUDI ARABIA

## Abstract

Whether the vaccine adjuvant potential of acute exercise is uniform among different populations, e.g., inactive persons, is unknown. This meta-analysis examines influenza vaccine antibody responses and the effect of physical activity, acute exercise, and their interaction. Inclusion criteria comprised randomized controlled trials with acute exercise intervention and influenza vaccination antibody measurements at baseline and 4–6 weeks, and participant baseline physical activity measurement; there were no exclusion criteria. Searching via six databases (Medline, Embase, CINAHL, Scopus, Web of Science, and Physiotherapy Evidence) and two clinical registries (WHO and NIH), nine studies were identified and assessed with the Cochrane revised risk-of-bias tool. Data analysis comprised one-stage random-effects generalized linear mixed-effects models with random intercept. Seven of nine identified studies, all of high risk of bias, provided data for 550 included participants. Clinical measures of antibody response tended to be higher in the acute-exercised participants compared to rested controls and physically active compared to inactive. Physical activity significantly increased H1 strain seroconversion (adjusted odds ratio (aOR) 1.69, 95%CI: 1.02–2.82) among all participants and titer response (aOR 1.20, 95%CI: 1.03–1.39) among the acute exercise group. Increasing age frequently reduced immunogenic responses whereas body mass index and sex had little-to-no effect. Adjuvant effects were more pronounced with interventions exercising the same arm in which the vaccination was administered. H1 response was increased by both physical activity and the acute exercise-physical activity interaction. Given the observed modifications by age and the subset analysis suggesting the benefit is more pronounced in older populations, future attention is due for acute exercise-PA interactions to impact vaccination response in the at-risk population of older adults. Further, we identify localized exercise as the likely most-effective protocol and encourage its use to augment the available evidence.

## Introduction

Globally, seasonal influenza causes between 290,000 and 650,000 deaths annually [1]. The seasonal influenza vaccination is one preventative tool but it its effectiveness is only 30–60% [2]. A further concern is that vaccinations are often less effective in at-risk populations, such as persons with obesity [3], and these populations are growing [4].

Regular sufficient accumulation of physical activity (PA), for example through chronic exercise, confers benefits of reduced risk of chronic health conditions [5, 6]. Evidence suggests being physically active or chronic exercise interventions have positive effects on the immune system, including vaccination responses [7–12]. PA is known to reduce chronic inflammation as well as reduce adiposity and increase muscle mass; these changes are thought to lead to the improved immune responses as seen after vaccination [13]. Improved vaccination response has also been shown following a single bout of moderate-intensity exercise (acute exercise) [9, 14, 15]. Acute exercise causes lymphocytosis and increased myokines which have been hypothesized to increase the efficiency and efficacy of antigen uptake and presentation [13]. This improved response may be most apparent in those with low immunogenic responses [16]. This is of particular concern in the present context as PA levels appear to decrease in response to confinement measures taken to combat the spread of COVID-19 which, like influenza, disproportionately affects mortality rates for at-risk persons [17]. Although several studies have examined the effect of acute exercise on vaccination responses, none have examined if this effect differs between physically active and inactive populations nor has any meta-analysis considered the potential interaction effect of acute exercise and PA on vaccination responses. The relationship between PA and risk of noncommunicable disease or premature mortality is nonlinear with greatest risk reduction apparent with increases in PA at lower PA levels [18]; therefore, it may follow that benefits on vaccination response from an acute exercise adjuvant are most pronounced in inactive populations. A better understanding of the acute exercise-PA relationship to vaccination response could have implications for vaccination programs, such as dosing variations or administration protocols which include a pre-vaccination acute exercise component, particularly where resources are limited or urgent protection is needed. Therefore, we investigated whether immunogenicity varied with acute exercise versus rested control, sufficient versus insufficient PA, or interactions between these. We further reviewed and analyzed the effect of different acute exercise prescriptions as this has been hypothesized to explain differences in efficacies of interventions [19]. We found some benefit from PA and interaction effects on antibody response.

## Methods

The relationship between immunogenicity and acute exercise-PA was limited to influenza vaccination as this was the most frequently used vaccination in the acute-exercise literature. Immunogenicity outcomes included seroconversion, seroprotection, and change in antibody titer from baseline to 4–6 weeks post vaccination. No population limits were employed; we considered age, sex, and body mass index (BMI, as a measure of body composition) as potential moderators because of suggestions that immunosenescence, sex, and obesity may alter vaccination response [15]. We evaluated strain-specific responses because results in the literature have shown inconsistent effects of acute exercise by strain variant and strains are known to exhibit different degrees of immunogenicity. This strain-specific approach allows greater capacity for generalizability to other contagions such as COVID-19 because of the difference in immunogenicity between influenza strains.

This study was deemed of negligible risk and exempt from ethical review by the University of Sydney Ethics Office.

### Outcome

The primary outcomes were the effect of acute exercise (single bout of <60 min) and of physical activity on influenza vaccination immunogenicity (i.e., seroconversion, seroprotection, and antibody titer response) at 4–6 weeks. The secondary outcome was the interaction effect on immunogenicity. Outcomes effects were measured as odds ratios.

### Search strategy, selection, assessment

Following consultation with an academic librarian, a systematic search (PROSPERO CRD42020166646) was conducted on 14 February 2020 (and rerun 12 March 2021 to confirm currency) to identify randomized controlled trials testing the effects of acute exercise on antibody response to influenza vaccination. We used six databases (Medline, Embase, CINAHL, Scopus, Web of Science, and Physiotherapy Evidence) and two clinical registries (WHO and NIH), and employed three search concepts: exercise, influenza, and antibodies. Inclusion criteria included randomized controlled trials with both antibody and PA measurements; there were no age, sex or language limits. Studies were screened and assessed using the Cochrane revised risk-of-bias tool [20] independently by two authors [EG and VL]. Authors of ten relevant studies were contacted to find out whether participant PA was measured at baseline and, in the affirmative (n = 9), whether non-identifiable participant-level data could be shared. Seven of nine studies shared data. (See S1 File Literature Review for full search strategy and bias assessment).

### Data

Requested data included de-identified participant baseline measures (sex, age, PA, BMI, and antibody titer), intervention/control status, and antibody titer at 4–6 weeks’ follow-up. The data were pooled from the seven studies characterized in Table 1 and presented briefly here. Healthy adult subjects were recruited and randomized by sex to control or acute exercise conditions. Baseline questionnaires collected demographics, including self-reported PA. Subjects underwent the acute exercise (n = 382) or rested control (n = 168) condition and received a vaccination administered to their deltoid. Data were checked for accuracy with published reports and no issues found.

**Table 1:** Study descriptions.

Study author, year [reference]	Participants (included n of total n; % male; age range in years (median, IQR))	Acute exercise		Acute exercise benefit to antibody response (antibody measure timing)
		condition (n, where subgroups exist)	timing relative to vaccination (n, where subgroups exist)	
1, Campbell, 2010 [21]	156 of 156; 49%; 18–32 (20, 19–21)	Two upper body eccentric movements at 85% 1RM for ~25min.	Immediate (38), 6 h (39) or 48 h (39) prior to vaccination	No effect (0-4wk)
2, Edwards, 2010, [16]	159 of 160; 50%; 18–35 (20, 20–21); adapted from Whitehall II	Two upper body eccentric movements at 60% (40)-85% (40) or 110% (39) 1RM for ~25min.	Immediately prior	Increased response in B strain and, in males, H3 (0-4wk)
3, Edwards, 2007, [22]	56 of 60; 45%; 18–31 (20, 19–21)	Two upper body movements at 85% 1RM for ~25min.	Immediately prior	Increased response in females (0-6wk)
4, Edwards, 2006, [23]	40 of 60^a^; 45%; 19–33 (21, 20–23)	45min cycling comprising incremental ergometer test followed by 4min active recovery and 25min at 55%max workload.	Immediately prior	Increased response in females (0-4wk)
5, Bohn-Goldbaum, 2019, [24]	45 of 47; 49%; 65–87 (73, 67–78)	Upper and lower body resistance exercises (5 movements-each 3 sets of 8 reps) at 60% 1RM for a total of 45min.	Immediately prior	No effect (0-4wk)
6, Lee, 2021, [19]	68 of 78; 62%; 18–30 (22, 20–25)	Upper (32) or lower (20) body resistance exercise comprising 5 reps of 3 moderate intensity movements for ~15min.	Post vaccination (18 from upper body group); all others immediately prior vaccination	Increased response in B strain (0-4wk)
7, Elzayat, 2020, [25]	26 of 29; 35%; 65–84 (72.5, 69.8–74.3)	Upper body exercise comprising 10 sets of 5 reps of 2 movements at 80% 1RM for ~25min.	Immediately prior	No effect (0–6 wk)

Study characteristics describe only those participants who underwent an acute exercise intervention or control condition and had a follow-up titer measure for at least one strain; the total n refers to the recruited sample size.

^a^Study included participants in a non-relevant intervention condition (n = 20).

The acute exercise bout comprised 15–50 minutes resistance (n = 359; studies 1, 2, 3, 5, 6 and 7 [16, 19, 21, 22, 24, 25]) or aerobic exercise (n = 20; study 4 [23]). Most exercise bouts were conducted immediately prior vaccination but for two exercise groups this occurred six or 48 hours prior (n = 39 for each group; study 1 [21]) and for one test group this occurred directly following vaccination (n = 16; study 6 [19]). In all studies, control groups sat quietly for an equivalent amount of time as the exercise task.

Blood samples were collected at baseline and 4–6 weeks after vaccination, the time frame for peak antibody development [26]. In all studies, serum samples were later analyzed for anti-influenza antibody titers using hemagglutination inhibition assays. Our analysis was limited to individuals who had a titer for at least one strain at follow-up. This was 96.5% of all individuals (study range: 87.1–100%).

Self-reported PA was measured by five separate tools across the studies: the Physical Activity Scale for Elderly (PASE) [27], the Godin-Shephard Leisure-Time Physical Activity Questionnaire [28], a questionnaire adapted from the Whitehall II study [29], or an open-ended question. For each tool, a dichotomous variable for sufficient PA attainment was computed. The World Health Organization recommended amount of PA for adults is 2.5 hours/week moderate PA (MPA) or 1.25 hours/week vigorous PA (VPA) or a combination thereof [30]. Where necessary, we applied time cut-offs or lower intensity classifications to prevent falsely categorizing participants as meeting recommendations (e.g., a response category of 2–3 hours/week MPA would include insufficient PA and so this response was coded as inactive). For study 1 [21], the data was recorded as total minutes per year of sweaty activity; the equivalent of 2.5 hours/week was considered sufficiently active. For studies 2–4 [16, 22, 23], available data was categorical; sufficient PA was defined as moderate or vigorous PA of at least 3 hours/week or at least 1 hour each of vigorous and moderate PA. For PASE measurements (study 5 [24]), available data was recorded as total kcal/week expended through MPA, VPA, stairs, walking and weightlifting. Sufficient PA is equivalent to 1125 kcal/week through MPA alone or 750 kcal/week through VPA alone. Therefore, the cut-off for sufficient PA was set at ≥1125 kcal/week in order to minimize including recorded expenditure due to light PA or weight training. For study 6 [19], responses were computed as moderate-to-vigorous PA units following the protocol of Godin [28], with a cut-off of ≥24 units (substantial benefits) as sufficient PA. For study 7 [25], cardiovascular activities were classified as moderate or vigorous intensity, or neither, according to Center for Disease Control and Prevention criteria [31], and converted to hours/week using recorded frequency responses; rehabilitative exercises (n = 3) were excluded from classification.

### Analysis

ANOVA, Kruskal-Wallis, and chi-square tests were used to compare group demographic characteristics.

The antibody response was evaluated separately by H1, H3, and B strain variants. To avoid double-counting individuals, only the primary B strain measurements were used where quadrivalent vaccine was administered (studies 6 and 7 [19, 25]; all other studies used trivalent vaccines). Antibody response was characterized dichotomously for seroprotection and seroconversion, with seroprotection defined as a titer ≥40 units and seroconversion as titer increases of four-fold or greater (using values of 5 where titer measurements were <10) [32]. Antibody response was also evaluated on a continuous measure of the difference from baseline to follow-up of titer values which were logged to account for skewness.

To examine the relationships between acute exercise, PA and antibody outcomes (seroconversion and titer change), we conducted one-stage random-effects individual participant data (IPD) meta-analyses using generalized linear mixed-effects models with random intercept with alpha set at 0.05 (lme4 package for R [33]). This approach allows for flexible modelling of within and between study variation [34]. Heterogeneity was measured by I^2^. The first model compared the effect of the acute exercise intervention on titer change in all participants and separately among physically active and inactive participants. Model two compared the effect of being physically active or inactive on titer change in all participants and in the acute exercise intervention and control participants. Models three and four repeated these comparisons but used the seroconversion odds ratio outcome. All models were also adjusted for age, sex, and BMI.

Because of the mixed-age composition in the data, sensitivity analyses were then conducted on the titer change outcome by repeating the models while restricting participants to those under 36 years-old (i.e., omitting studies 5 and 7, [24, 25]). A similar analysis with just those over 65 years of age was preferred because of implications related to immunosenescence but was not possible to perform due to limited sample size. (Available data did not include any participants 36–64 years-old.) Age-sensitivity analysis was attempted with the seroconversion outcome but not possible due to the high proportion of seroconverted participants and the small numbers of inactive participants. The sample size was also too small to get valid results for any modelling of acute exercise-PA effects on seroprotection.

Included studies used both aerobic and resistance exercise protocols. One hypothesis for the mechanism of the adjuvant event effect of acute exercise is that immune cells are primed to by the localized muscular damage of resistance exercise to target that location [35]. Following this hypothesis, results could differ by intervention method. Therefore, a sensitivity analysis was conducted on titer change outcomes by repeating the models with only rested controls and participants who performed resistance exercise in the arm receiving the vaccination (“same-arm” group). This excluded 20, 20 and 7 exercised-participants from studies 4, 6 and 7 [19, 23, 25], respectively.

## Results

### Literature and demographics

**Fig 1:**
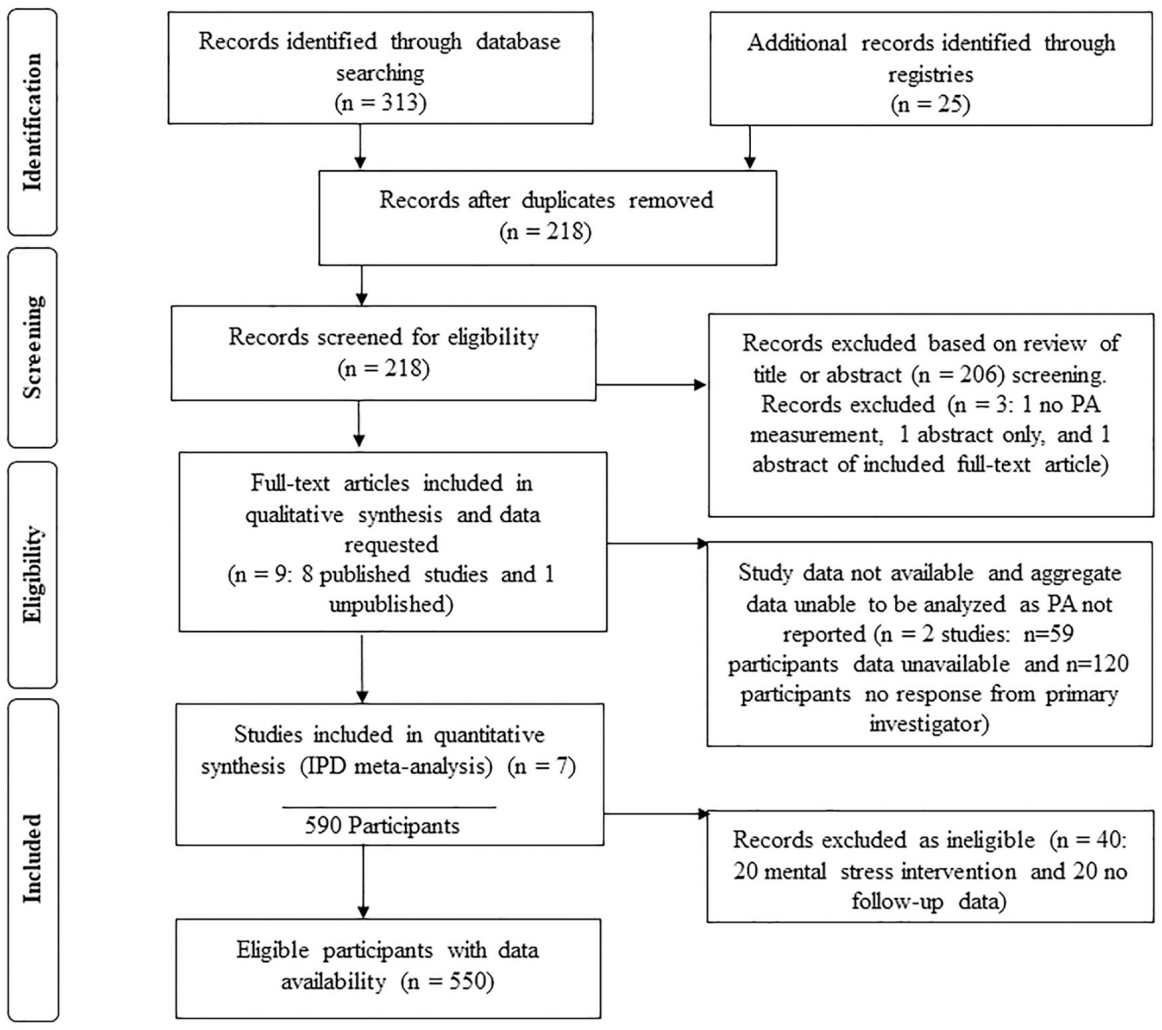
PRISMA flowchart of study and participant selections.

The literature search identified 12 potential studies following screening. Excluded were two studies because PA was not measured (n = 1) or full data had not yet been analyzed (n = 1), while a third study was an abstract using data from an already-included study [24]. The nine studies investigating acute exercise and influenza vaccination antibodies and which measured PA (Fig 1) were judged to have high risk of bias (and therefore constitute low quality of evidence) primarily due the randomization process being insufficiently described or concealed prior to assignment and the inherent inability to blind exercise interventions. Another factor was lack of explanation for missing data. (See S1 File for literature review and S2 File for forest plots).

Seven of these studies provided data and are described in Table 1, with data collection and processing detailed in S1 File. Interventions included both aerobic (n = 1 study) and resistance (n = 6 studies) exercises and ranged in duration (15–45 min). While most protocols delivered vaccination immediately following the exercise intervention, the time from intervention to vaccination ranged 48 hours prior to 6 hours post.

We limited analysis to participants providing at least one follow-up titer strain. Thus, our sample comprised 550 of 570 (96.3%) eligible participants, as described in Table 2, with no differences in characteristics between participant groups.

**Table 2:** Study participants’ characteristics.

	Participant Groups				
	Control -inactive	Control -physically active	Acute exercise -inactive	Acute exercise -physically active	Total (n = 550)
**N (% sufficiently active)**	57 (0%)	104 (100%)	144 (0%)	233 (100%)	337 (62.6%)
**Age in years, median (IQR)**	21.0 (20.00–66.25)	21.0 (19.00–24.25)	21.0 (20.00–23.00)	20.0 (19.00–23.00)	21.0 (19.00–24.00)
**Sex (female)**	31 (54.4%)	53 (51.0%)	76 (52.8%)	111 (47.6%)	276 (50.2%)
**BMI in kg/m**^**2**^, **median (IQR)**	23.7 (21.72–26.91)	23.3 (21.25–25.81)	22.6 (20.96–24.98)	22.7 (20.99–24.54)	22.8 (21.05–25.10)
**H1 baseline GMT (SD)**	26.5 (4.51)	23.1 (3.57)	27.3 (3.63)	21.9 (2.92)	23.9 (3.38)
**H1 1-month GMT (SD)**	359.9 (7.46)	395.5 (5.35)	369.8 (4.33)	413.2 (4.70)	393.2 (4.90)
**H3 baseline GMT (SD)**	28.1 (4.51)	41.8 (3.87)	24.4 (2.97)	44.1 (3.83)	35.1 (3.70)
**H3 1-month GMT (SD)**	166.0 (4.30)	241.1 (3.37)	158.8 (4.27)	242.6 (3.33)	206.3 (3.74)
**B baseline GMT (SD)**	33.4 (4.06)	42.5 (4.77)	76.4 (5.60)	44.6 (4.40)	49.5 (4.83)
**B 1-month GMT (SD)**	196.7 (6.52)	343.1 (4.23)	441.7 (4.84)	291.8 (4.21)	326.7 (4.65)

Values are expressed as n (%) for categorical variables and median (interquartile range) or geometric mean (geometric standard deviations) for continuous variables. Sample sizes may vary due to missing data. Multi-group comparisons indicated no group differences at p < .05 in age, BMI or proportion female. H1, H3, and B refer to the three influenza strains. GMT = Geometric mean titer.

**Fig 2:**
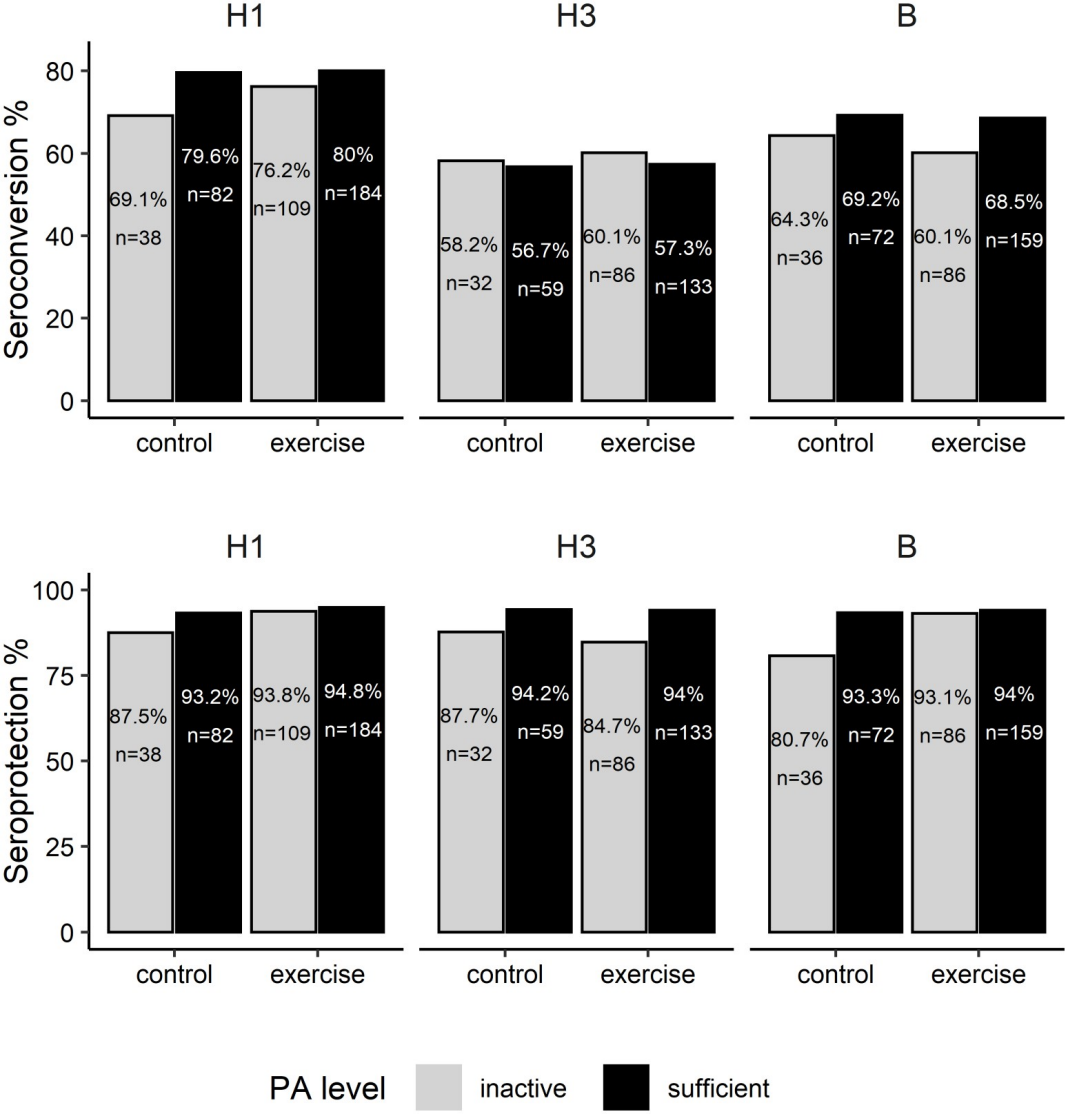
Seroconversion and seroprotection rates. Seroconversion and seroprotection rates at one month following influenza vaccination among acute exercise and control participants, by strain, physical activity and acute exercise groups.

Overall, seroprotection rates for all strains were high while seroconversion rates were less pronounced, as shown in Fig 2. Both clinical measures tended to be higher in physically active compared to inactive participants and in exercised participants compared to controls.

### Titer change and acute exercise effects

The effect of the acute exercise intervention on the increase in antibody titer did not differ for any strain in either the adjusted or unadjusted models among the entire data set or any subgroup (Table 3 and S2 File).

**Table 3:** The effect of acute exercise on influenza H1 strain titer change from baseline to follow-up.

Predictors	OR (95%CI)	p	aOR (95%CI)	p
	**all participants**			
**Exercise (control)**	1.04 (0.92–1.17)	0.53	1.04 (0.92–1.18)	0.50
**Age (year)**			0.98 (0.97–0.99)	<0.01^a^
**Sex (male)**			1.04 (0.93–1.17)	0.46
**BMI (kg/m** ^**2**^ **)**			1.00 (0.99–1.02)	0.67
**I** ^**2**^	0.29		0.08	
**Observations**	543		539	
	**physically active participants**			
**Exercise (control)**	1.14 (0.97–1.33)	0.10^a^	1.14 (0.97–1.33)	0.10^a^
**Age (year)**			0.98 (0.97–1.00)	0.02^a^
**Sex (male)**			1.05 (0.92–1.22)	0.47
**BMI (kg/m** ^**2**^ **)**			1.01 (0.98–1.03)	0.57
**I** ^**2**^	0.29		0.09	
**Observations**	333		331	
	**inactive participants**			
**Exercise (control)**	0.90 (0.72–1.13)	0.36	0.89 (0.70–1.10)	0.30
**Age (year)**			0.98 (0.97–0.99)	<0.01^a^
**Sex (male)**			1.06 (0.88–1.28)	0.55
**BMI (kg/m** ^**2**^ **)**			1.01 (0.98–1.04)	0.52
**I** ^**2**^	0.27		0.03	
**Observations**	198		196	

The effect of acute exercise on influenza H1 antibody titer change by physical activity: linear regression (unadjusted and adjusted modelling) on change in antibody titer levels. Estimates are given with 95%CI in parentheses. Predictors are by unit increase (age and BMI) or versus comparator (exercise and sex). BMI = body mass index.

^**a**^significant in same-arm analysis

Two sensitivity analyses were performed, with full models presented in S2 File and significant effects indicated in Table 3. In the sensitivity analysis using only the younger adults, no significant effect was found for acute exercise on antibody titers. In the sensitivity analysis using only same-arm exercising participants (and controls), however, the adjusted odds of higher H1 antibody response were significantly increased by acute exercise in the physically active group at 1.19 (95%CI 1.00–1.40, p = 0.041). No acute exercise effects were found in other strains in these analyses.

### Titer change and physical activity effects

The effect of PA on the increase in antibody titer was significant for the H1 strain only among participants who performed acute exercise (OR = 1.20, 95%CI: 1.03–1.39, p = 0.02; adjusted OR (aOR) = 1.20, 95%CI: 1.03–1.40, p = 0.02) (Table 4 and S2 File). There was a trend (p = 0.06) for a similar PA effect for the H1 strain for all participants. No effect of PA was seen in other groups or in the other strains.

**Table 4:** The effect of physical activity level on influenza H1 strain titer change from baseline to follow-up.

Predictors	OR (95%CI)	p	aOR (95%CI)	p
	**all participants**			
**PA (inactive)**	1.12 (0.99–1.27)	0.06^a^	1.12 (0.99–1.27)	0.07^a^
**Age (year)**			0.98 (0.97–0.99)	<0.01^a^
**Sex (male)**			1.05 (0.94–1.17)	0.41
**BMI (kg/m** ^**2**^ **)**			1.00 (0.98–1.02)	0.60
**I** ^**2**^	0.28		0.08	
**Observations**	531		527	
	**acute exercise participants**			
**PA (inactive)**	1.20 (1.03–1.33)	0.02^a^^,^^b^	1.20 (1.03–1.40)	0.02^a^^,^^b^
**Age (year)**			0.98 (0.97–1.00)	0.01^a^
**Sex (male)**			1.07 (0.94–1.23)	0.30
**BMI (kg/m** ^**2**^ **)**			1.01 (0.98–1.03)	0.65
**I** ^**2**^	0.27		0.07	
**Observations**	373		370	
	**control participants**			
**PA (inactive)**	0.96 (0.77–1.21)	0.71	0.95 (0.76–1.18)	0.63
**Age (year)**			0.98 (0.96–0.99)	<0.01^a^
**Sex (male)**			1.01 (0.82–1.23)	0.94
**BMI (kg/m** ^**2**^ **)**			1.01 (0.98–1.04)	0.67
**I** ^**2**^	0.30		0.30	
**Observations**	158		157	

The effect of physical activity (PA) level on influenza H1 antibody titer change by acute exercise: linear regression (unadjusted and adjusted modelling) on change in antibody titer levels. Estimates are given with 95%CI in parentheses. Predictors are by unit increase (age and BMI) or versus dichotomous comparator (PA level and sex). Odds ratios are given with 95%CI in parentheses.

^**a**^significant in same-arm analysis.

^**b**^significant in sensitivity analysis with younger population studies (<36 years-old).

The two sensitivity analyses were again performed, with full models presented in S2 File and significant effects indicated in Table 4. In both sensitivity analyses, effects were only seen in the H1 strain. The effect of PA on the H1 antibody titer in exercised participants remained significant when only young participants were included in the model (aOR = 1.20, 95%CI: 1.02–1.42, p = 0.03). In the same-arm analysis, PA significantly increased the odds for higher H1 levels (aOR 1.15, 95%CI:1.02–1.31, p = 0.03). The effect was seen in exercised participants (aOR 1.26, 95%CI:1.07–1.48, p<0.01) but not in rested controls.

### Seroconversion and acute exercise and PA effects

Acute exercise had no effect on ORs for seroconversion for any strain for all participants or sub-groups, in either the un-adjusted or adjusted models (S2 File).

In contrast, the meta-analysis revealed a significant effect of PA on H1 seroconversion in all participants (Table 5). The OR for H1 seroconversion among physically active participants was 1.73 (95%CI: 1.05–2.88); the direction of effect was stronger in the physically active acute exercise group (OR 1.79, 95%CI: 0.99–3.28) than in the physically active controls. When three potential moderators (sex, BMI and age) were included in the model, the increased odds for H1 seroconversion remained significant among all physically active participants (aOR 1.69, 95%CI: 1.02–2.82) but not among subgroups. PA had no effect on the odds of H3 or B strain seroconversion (S2 File).

**Table 5:** The effect of physical activity level on influenza H1 strain seroconversion.

Predictors	OR (95%CI)	p	aOR (95%CI)	p
	**all participants**			
**PA (inactive)**	1.73 (1.05–2.88)	0.03	1.69 (1.02–2.82)	0.04
**Age (year)**			0.96 (0.94–0.997)	0.01
**Sex (male)**			0.81 (0.51–1.30)	0.36
**BMI (kg/m** ^**2**^ **)**			1.00 (0.93–1.07)	0.89
**I** ^**2**^	0.55		0.17	
**Observations**	531		527	
	**acute exercise participants**			
**PA (inactive)**	1.79 (0.99–3.28)	0.05	1.74 (0.96–3.19)	0.07
**Age (year)**			0.97 (0.94–0.999)	0.02
**Sex (male)**			0.70 (0.41–1.20)	0.20
**BMI (kg/m** ^**2**^ **)**			1.00 (0.92–1.08)	0.93
**I** ^**2**^	0.37		0.08	
**Observations**	373		370	
	**control participants**			
**PA (inactive)**	1.47 (0.54–3.90)	0.43	1.27 (0.46–3.43)	0.62
**Age (year)**			0.95 (0.90–0.99)	0.03
**Sex (male)**			1.32 (0.54–3.32)	0.54
**BMI (kg/m** ^**2**^ **)**			1.01 (0.88–1.16)	0.91
**I** ^**2**^	0.73		0.35	
**Observations**	158		157	

The effect of physical activity (PA) level on influenza H1 antibody seroconversion by acute exercise: linear regression (unadjusted and adjusted modelling) on seroconversion. Estimates are given with 95%CI in parentheses. Predictors are by unit increase (age and BMI) or versus dichotomous comparator (PA level and sex). Odds ratios are given with 95%CI in parentheses.

### Moderators

Three potential moderators were included in the model: sex, BMI and age, as shown in S2 File. Sex had no effect on outcomes for any strain. BMI had no effect on H1 or B strains, but in the H3 strain increasing BMI predicted smaller change in the titer. This relationship was non-significant among population subsets, although there was a trend for this association in the acute-exercised physically active. Age, in contrast, frequently exerted a significant effect on the antibody response. For both H1 and B strains, increasing age consistently modified titer change predictions in a negative direction. For H3, the effect was not always statistically significant but worked in the same direction. A similar pattern held by strain for seroconversion, with increasing age lowering the aOR for seroconversion significantly in H1 and B strains. The age-related sensitivity analysis showed that when older (over 65 years) adults were removed from the sample, age did not exert any significant effect on titer change.

## Discussion

To better understand the role of exercise in vaccination responses, this study sought to determine whether influenza vaccine immunogenicity varied with acute exercise as an acute adjuvant, with a physically active lifestyle, or with interaction between these. This is the first study to evaluate the interaction between acute exercise and underlying physical activity behavior. We employed one-stage random-effects IPD meta-analyses using generalized linear mixed-effects models on antibody response among healthy adults, 18–87 years-old, from seven studies. We found selected benefit by PA in the H1 strain seroconversion, with sufficient PA increasing the odds to 1.69 (95%CI: 1.02–2.88, p = 0.040) for H1 seroconversion. In agreement with the literature [9], our data also showed some support for benefits from either acute exercise or PA on antibody titer levels and, through subset analysis, suggest the benefit is less pronounced in younger populations. Our large sample size (n = 550) lends strength to our findings and the inclusion of seven different influenza vaccines increases its power and ability to avoid anomalies due to a particular vaccine composition, which may explain some of the heterogeneity between studies.

Our findings imply public health bodies consider measures for communicable disease protection which also enable PA practice, as a possible mechanism to improve antibody response to vaccines. Additional subset analysis using only same-arm participants showed a stronger effect of acute exercise on H1 antibody titer increase in all same-arm participants and in physically active same-arm participants relative to controls than in the full analysis. Resistance exercise can cause cell damage in the exercised muscle; this damage can initiate the release of local danger signals which have been proposed to act as adjuvants, stimulating antigen-presenting dendritic cells and an adaptive immune response [36, 37]. The observed stronger effect in the same-arm participants is consistent with local danger signals playing a role. Further, because the same-arm acute exercise protocol elicited a greater effect, this may be a preferable intervention for future research into exercise as a vaccine adjuvant and afford opportunity to investigate the underlying mechanisms.

Contrary to our hypothesis, inactive persons did not benefit more from acute exercise as an adjuvant to influenza vaccination than active participants. Seroconversion rates were slightly higher in the physically active compared to inactive group for H1 and B strain variants, but they were lower for the H3 strain (57.1% vs 59.6%). In this strain, baseline titers among the physically active compared to inactive participants were higher (GMT of 43.3 vs 25.4, respectively, Table 2). One possibility is that the higher titer of H3 antibodies in the physically active has a limited potential to increase by four-fold when revaccinated. Antibody maintenance with PA has been previously demonstrated [38], and pre-immunization antibody titer levels have been shown to be inversely related to seroconversion [39], consistent with this suggestion. Another study observed non-seroconverters for the H3 strain had higher median PA scores but did not report baseline H3 titers [40], limiting the ability to draw inference.

Our sample comprised mainly young adults who met PA recommendations and this composition may be the reason we observed limited benefit from PA, in line with a null finding in a similar population [41]. Our model identified a significant difference from PA on H1 seroconversion, with physically active participants having 1.69 times the odds of seroconversion compared to inactive participants. The model also identified improved H1 titer response with PA and this was significant among acute-exercised participants. Other studies have also found variations in response by strain. For example, higher peak antibody titer responses have been demonstrated for both influenza A strain variants in elite athletes compared to non-athlete peers [12]. Higher peak antibody titer responses for the H1 strain, with a trend for the same for B, were found in adults with higher vigorous PA [42]. Higher responses have also been shown for H3 and sometimes B strains, but not for H1, in physically active older adults compared to sedentary or inactive peers [10, 11, 43, 44].

Deterioration with aging in the immune response to vaccination is well-documented [15, 45]. We observed that age lessened the antibody response in all models for the H1 and B strains. When we explored the models among the younger (< 36 years) participants only, we found a null result for nearly all moderators (e.g., BMI, sex and age). Improved response with PA is more consistently identified among older populations [11, 43, 44]. This suggests the young have the ability to respond well to vaccination regardless of sufficient PA; in contrast, with immunosenescence health factors such as being sufficiently physically active have a more pronounced effect. We were unable to separately investigate antibody response with just the older (≥65 years) participants due to their small sample size. Investigation of a potential acute exercise-PA interaction effect on immune response within older adults would be worthy of future exploration, particularly as older adults stand to benefit more from any adjuvant effect due to innate immunosenescence.

Antibody response to influenza vaccination in adults tends to be stronger in females [15]. Previous research indicates no sex difference in antibody response with respect to chronic exercise and no consistent sex difference with respect to acute exercise [9]. Therefore, the lack of sex-effect here is unsurprising.

In our findings one strain (H3) indicated higher BMI was associated with a smaller titer response among all participants. This was not significant among the inactive group, but a trend remained among physically active participants indicating inactivity may be a stronger moderator than BMI. The literature is mixed on the effect of obesity on antibody response but consistent on the association of obesity with reduced immune function [15]. We were unable to more closely investigate the moderating effects of obesity due to the small sample of people with obesity (5.2%) in our study. Given the increasing rates of obesity in developed countries and the obesity-associated higher risk of mortality and disease severity, future exploration of antibody responses in obese persons with respect to an acute exercise-PA interaction effect may produce findings of significance for public health approaches. Finally, our results contribute to the knowledge base surrounding mechanisms underlying the potential for enhanced immunogenicity through acute exercise and physical activity.

### Limitations

Our search strategy was inclusive and wide-reaching, however the sample size achieved only three-quarters of potential participant data. (We note that both studies not included in analysis found no significant overall effect from acute exercise.) The effect of our strategy also meant we included wide ranges of study interventions and adult ages, impacting the certainty of evidence. We were able to explore these variations in sub-analyses, however the lack of any participants 36–65 years-old should be identified as a limitation. Data with a full age set would aid further investigation.

Strengths in our study include that our sample comprised 62.8% of participants deemed sufficiently active which is comparable to the proportion meeting PA guidelines in high-income countries such as our sample [46] and that it was gender balanced.

We were unable to separately analyze acute aerobic and resistance exercise interventions, analyses which could lead to a better understanding of the most effective exercise interventions.

Finally, data for this meta-analysis came from a small number of studies with high risk of bias. This risk was due in part to the nature of exercise intervention studies (i.e., an inability to blind participants to intervention status) and sometimes in omission of protocol details (e.g., in [24] concealment details were not published). Nevertheless, some caution is warranted with our findings given the potential bias.

## Conclusion

Sufficient PA had a positive effect on the H1 immune response to influenza vaccination, increasing the odds to 1.69 (95%CI: 1.02–2.82, p = 0.040) for H1 seroconversion. Though we found some benefit from either acute exercise or PA on antibody titer levels, our finding did not support our hypothesis: there was no added benefit of acute exercise to inactive participants. Our findings point to new directions for exploration as subset analysis suggests acute exercise-PA interaction effects on immune response may be more pronounced in older populations. Such investigation has potential to identify means of improving antibody response for vaccines, particularly those for diseases which disproportionately affect older persons.

## Supporting information

S1 File Literature review. This supplement supports the literature search and assessment of data quality.
(DOCX)

S2 File Analyses. This supplement includes forest plots and additional models.
(DOCX)

S1 Data IPD data. (CSV)

S1 Checklist (DOCX)
