# Supplementary material for: Physical activity and acute exercise benefit influenza vaccination response: A systematic review with individual participant data meta-analysis
Source: PLoS One. 2022 Jun 15;17(6):e0268625. doi: 10.1371/journal.pone.0268625 (PMC9200169; doi:10.1371/journal.pone.0268625)
Supplement: S1 File — This supplement supports the literature search and assessment of data quality. (DOCX) [file pone.0268625.s001.docx]

# S1 Literature review

| Section | Content | Page |
| --- | --- | --- |
| I | Literature searchTable S1. Characteristics of studies included in the literature review | 2 |
| II | Quality assessmentTable S2. Risk of Bias | 5 |
| III | References | 7 |

# Section I. Literature search

The PROSPERO search (CRD42020166646) was lodged 6 Feb 2020.

## Databases and registries

Databases and registries included Medline, Embase, CINAHL, Scopus, Web of Science, and Physiotherapy Evidence databases, and WHO and clinicaltrials.gov registries.

## Search strategy and terms

The search strategy was designed to identify influenza vaccination studies employing acute exercise as a stressor. Further inclusion criteria were full text article (published full text or published abstract with full draft paper completed) or study at analysis stage with full draft completed (if registered trial) of a randomized controlled trial with antibody outcome measures.

The search was conducted 14 February 2020 included works published (or registered) through 31 January 2020. The search was rerun 12 March 2021 to confirm currency of the literature; no new studies were identified in the rerun.

The Medline search strategy was as follows:

Exercise/ OR exercis*.mp. OR Walking/ OR Walk*.mp. OR exp Running/ OR running.mp. OR Resistance Training/ OR resistance training.mp. OR exp Exercise Test/ OR exercise test*.mp. OR exp Physical Fitness/ OR Physical fitness.mp.) AND (Influenza, Human/ OR influenza*.mp. OR Influenza Vaccines/ OR influenza vaccin*.mp. OR influenza Vaccination*.mp. OR exp Influenza A virus/ OR influenza A Virus*.mp. OR Influenza B virus/ OR influenza B Virus*.mp.) AND ((titer* or titer*).mp. OR Antibodies, Viral/ OR Viral antibod*.mp. OR exp Antibody Formation/ OR Antibody Formation.mp. OR exp immunity, active/ or immunity, humoral/ or immunogenicity, vaccine/).

The change database function was then employed to run the search in Embase and CINAHL.

The Scopus search was as follows:

( ( KEY ( influenza ) OR TITLE-ABS-KEY ( influenza W/3 vaccin* ) OR TITLE-ABS-KEY ( influenza W/3 virus* ) ) AND ( TITLE-ABS-KEY ( physical AND exertion ) OR TITLE-ABS-KEY ( exercis* ) OR TITLE-ABS-KEY ( resistance W/3 train* ) ) AND ( TITLE-ABS-KEY ( antibod* ) OR TITLE-ABS-KEY ( viral W/3 antibod* ) OR TITLE-ABS-KEY ( form* W/3 antibod* ) OR TITLE-ABS-KEY ( vaccin* W/3 response ) OR TITLE-ABS-KEY ( humo*ral W/3 response ) ) ) AND ( LIMIT-TO ( EXACTKEYWORD , "Human" ) OR LIMIT-TO ( EXACTKEYWORD , "Humans" ) ).

Web of Science search was with “all fields” selected for “Exercis* AND vaccine* AND influenza AND antibod*”; while “Exercis* vaccine* influenza” was used in the Physiotherapy Evidence Database and “exercise influenza vaccine” in the WHO (https://apps.who.int/trialsearch/) and clinicaltrials.gov registries.

## Screening

The screening and selection process is depicted in Figure 1 in main manuscript. Titles and abstracts of 218 references were screened by EG and VL for inclusion criteria* using the Covidence app, with 96% initial agreement. Discussion between the two co-authors led to 100% agreement without a third author consultation.

Inclusion criteria: RCT, any acute exercise intervention, influenza vaccination, antibody response between 4-6weeks; human participants; no limits on age, gender; no language limits. Exclusion criteria: all exclusion criteria at the initial screening stage paralleled inclusion criteria (e.g., study design other than an RCT was a basis for exclusion).

Twelve studies were identified as eligible for possible inclusion. Authors were contacted for a second screening stage to confirm a baseline physical activity measurement was made and, for abstracts/registries, the study had progressed to analysis of the full data. In the affirmative, the study was deemed eligible and individual data was requested. *In practice, screening was most efficient by first eliminating articles which contradicted inclusion criteria. For example, where a study title contained “position statement on” (i.e., not an RCT) or “in mice” (i.e., not human participants), the study was excluded.

## Search results

### Included Studies

We included in the literature review 9 RCT published since 2006 [1-9], as described in S1 Table. Sample sizes ranged from 29 [9] to 160 [7]. Five were conducted in the UK [1, 4-7], two in Australia [3, 8] and two in the US [2, 9]. Participants were 18 to 87 years old, with males comprising 35%-62% of each study.

Interventions included resistance training in the vaccinated arm [3-5, 7-9] or contralateral arm [9] or legs [8]. One study used cycle ergometry [6] and two used walking [1, 2]. Two studies measured outcome at 6 weeks [5, 9] with the remainder measuring at about 1 month [1-4, 6-8].

### Excluded studies

Three studies were excluded from the literature review. The primary investigator for one study [10] stated physical activity was not measured, thus this was excluded. A second study (intervention n=11, control n=12) had not progressed beyond abstract stage [11] and did not respond to further enquiry regarding study progress and data sharing. A third exclusion was an abstract [12] of a full study [3] already included.

## Study characteristics

Study characteristics were obtained from published methodology or pre-publication platforms, with details supplied by study authors where the PA measurement methodology was unstated.

## Individual data collection process and items

Study authors or primary investigators were contacted to request data. As previously noted, two studies declined to share data [1, 2].

Requested items included method of measurement for physical activity and de-identified participant characteristics (physical activity measure, age, sex, height and weight or body mass index) and antibody titers.

De-identified data were kindly shared in spreadsheet format; antibody measures were generally shared as mean raw titer.

Provided data were checked against published (or pre-published) reports, checking sample size and demographics, and confirming mean antibody direction of change.

## Table S1. Characteristics of studies included in the literature review

| Study author, year |  | **Acute exercise** |  | **Influenza strains** |
| --- | --- | --- | --- | --- |
|  | N (% male), age range (years) | **condition** | **timing relative to vaccination (n, where subgroups exist)** | **(H1, H3, B)** |
| Campbell, 2010 [4] | 156 (49%), 18-32 | Two upper body eccentric movements at 85% 1RM for ~25min. | Immediate (38), 6 h (39) or 48 h (39) prior to vaccination | A/Solomon Islands/3/2006, A/Wisconsin/67/2005, B/Malaysia/2506/ 2004 |
| Edwards, 2010 [7] | 160 (50%),18-35 | Two upper body eccentric movements at 60%, 85% or 110% 1RM for ~25min. | Immediately prior | A/Brisbane/59/2007, A/Uruguay/716/2007, B/Florida/4/2006 |
| Edwards, 2007 [5] | 60 (45%),18-31 | Two upper body movements at 85% 1RM for ~25min. | Immediately prior | A/New Caledonia/20/99, A/Wyoming/3/2003, B/Jiangsu/10/2003 |
| Edwards, 2006 [6] | 60 (53%),19-33 | 45min cycling (incremental ergometer test, 4min active recovery, then 25min at 55%max workload) | Immediately prior | A/New Caledonia/20/99,  A/Panama/2007/99,  B/Shangdong/7/97 |
| Bohn-Goldbaum, 2019 [3] | 47 (49%),65-87 | Upper and lower body resistance exercises (5 movements-each 3 sets of 8 reps) at 60% 1RM for a total of 45min. | Immediately prior | A/California/7/2009, A/Perth/16/2009, B/Brisbane/60/2008 |
|  |  |  |  |  |
| Lee, 2021 [8] | 78 (62%),18-30 | Upper or lower body resistance exercise comprising 5 reps of 3 moderate intensity movements for ~15min. | Post vaccination (18 from upper body group); all others immediately prior vaccination | A/Singapore/GP1908/2015, A/ Hong Kong/4801/2014, B/Brisbane/60/2008, BPhuket/3073/2013 |
| Elzayat, 2021 [9] | 29 (35%),65-84 | Upper body exercise comprising 10 sets of 5 reps of 2 movements at 80% 1RM for ~25min. | Immediately prior | A/Michigan/45/2015, A/Singapore/INFIMH-16-0019/2016, B/Colorado/06/2017, B/Phuket/3073/2013 |
| Long, 2012 [1] | 120 (50%),18-30 and 50-64 | Walking at >55% age-predicted maximum heart rate for 45min. | Immediately prior | A/California/7/ 2009, A/Perth/16/2009, B/Brisbane/60/2008 |
| Ranadive, 2014 [2] | 59 (41%), 55-75 | Treadmill walking at 55-65% max heart rate for 40 min. | Immediately prior | A/California/7/2009, and A/Perth/16/2009, B/Brisbane/60/2008; or A/California/7/2009, A/Victoria/210/2009, B/Brisbane/60/2008 |

# Section II. Quality assessment

## Risk of bias assessment outcome

The Cochrane revised risk-of-bias tool was applied at the outcome level. The overall risk of bias was deemed high, as shown in Table S2. Concerns for randomization were generally due to the randomization process being insufficiently described [1, 2, 4, 6, 7] or concealed [5, 9] prior to assignment. Blinding is not possible for exercise intervention studies; thus, we deemed all studies high risk of bias for blinding. Missing antibody outcome data of 5% or more was generally unexplained [1, 5-7, 9] or missingness not reported [3, 8]. One study presented sex but not intervention effects for an antibody strain [2].

## Table S2. Risk of bias assessment

| Study # | Randomization process | Deviations from intended interventions | Missing outcome data | Measurement of the outcome | Selection of the reported result | Overall |
| --- | --- | --- | --- | --- | --- | --- |
| 1. Campbell, 2010 [4] | High | Low | Low | Low | Some concerns | High |
| 1. Edwards, 2010 [7] | High | Low | High | Low | Some concerns | High |
| 1. Edwards, 2007 [5] | High | Low | High | Low | Some concerns | High |
| 1. Edwards, 2006 [6] | High | Low | High | Low | Some concerns | High |
| 1. Bohn-Goldbaum, 2020 [3] | Low | Low | High | Low | Some concerns | High |
| 1. Lee, 2021 [8] | Low | Low | High | Low | Some concerns | High |
| 1. Elzayat, 2021 [9] | High | Low | High | Low | Some concerns | High |
| 1. Long, 2012 [1] | High | Low | High | Low | Some concerns | High |
| 1. Ranadive, 2014 [2] | High | Low | High | Low | High | High |
| **OVERALL** | **High** | **Low** | **High** | **Low** | **Concerning** | **High** |

Study numbers correspond to those in the full manuscript.

# III. References

1. Long JE, Ring C, Drayson M, Bosch J, Campbell JP, Bhabra J, et al. Vaccination response following aerobic exercise: Can a brisk walk enhance antibody response to pneumococcal and influenza vaccinations? Brain, Behavior, and Immunity. 2012;26(4):680-7. doi: 10.1016/j.bbi.2012.02.004.

2. Ranadive SM, Cook M, Kappus RM, Yan H, Lane AD, Woods JA, et al. Effect of acute aerobic exercise on vaccine efficacy in older adults. Medicine and Science in Sports and Exercise. 2014;46(3):455-61. doi: 10.1249/MSS.0b013e3182a75ff2.

3. Bohn-Goldbaum E, Pascoe A, Singh MF, Singh N, Kok J, Dwyer DE, et al. Acute exercise decreases vaccine reactions following influenza vaccination among older adults. Brain, Behavior, & Immunity - Health. 2020;1:100009. doi: <https://doi.org/10.1016/j.bbih.2019.100009>.

4. Campbell JP, Edwards KM, Ring C, Drayson MT, Bosch JA, Inskip A, et al. The effects of vaccine timing on the efficacy of an acute eccentric exercise intervention on the immune response to an influenza vaccine in young adults. Brain, Behavior, and Immunity. 2010;24(2):236-42. doi: 10.1016/j.bbi.2009.10.001.

5. Edwards KM, Burns VE, Allen LM, McPhee JS, Bosch JA, Carroll D, et al. Eccentric exercise as an adjuvant to influenza vaccination in humans. Brain, Behavior, and Immunity. 2007;21(2):209-17. doi: 10.1016/j.bbi.2006.04.158.

6. Edwards KM, Burns VE, Reynolds T, Carroll D, Drayson M, Ring C. Acute stress exposure prior to influenza vaccination enhances antibody response in women. Brain, Behavior, and Immunity. 2006;20(2):159-68. doi: 10.1016/j.bbi.2005.07.001.

7. Edwards KM, Campbell JP, Ring C, Drayson MT, Bosch JA, Downes C, et al. Exercise intensity does not influence the efficacy of eccentric exercise as a behavioural adjuvant to vaccination. Brain, Behavior, and Immunity. 2010;24(4):623-30. doi: 10.1016/j.bbi.2010.01.009.

8. Lee VY, Bohn-Goldbaum E, Fong J, Barr IG, Booy R, Edwards KM. Analgesic and adjuvant properties of exercise with vaccinations in healthy young population. Hum Vaccin Immunother. 2021:1-7. doi: 10.1080/21645515.2020.1859322.

9. Elzayat MT, Markofski MM, Simpson RJ, Laughlin M, LaVoy EC. No Effect of Acute Eccentric Resistance Exercise on Immune Responses to Influenza Vaccination in Older Adults: A Randomized Control Trial. Frontiers in Physiology. 2021;12(1279). doi: 10.3389/fphys.2021.713183.

10. Housel LA, Beltran TA, Spooner C, Collins LC, Jr., Ewing DF, Williams M, et al. A randomized controlled trial of NSAIDs or exercise to reduce delayed local pain after influenza vaccination. Journal of Allergy and Clinical Immunology: In Practice. 2021;9(2):1018-20.e1. doi: 10.1016/j.jaip.2020.08.058.

11. Colburn A, Wright S, Lopez V, Giersch G, Belval L, Hosokawa Y, et al. Aerobic exercise and environmental heat stress as adjuvants to seasonal influenza vaccine. FASEB Journal Conference: Experimental Biology. 2018;32(1 Supplement 1). doi:

12. Edwards KM, Pascoe AR, Fiatarone-Singh MA, Singh NA, Kok J, Booy R. A randomised controlled trial of resistance exercise prior to administration of influenza vaccination in older adults. Brain, Behavior, and Immunity. 2015;49 (Supplement 1):e24-e5. doi:
