# Supplementary material for: Physical activity and acute exercise benefit influenza vaccination response: A systematic review with individual participant data meta-analysis
Source: PLoS One. 2022 Jun 15;17(6):e0268625. doi: 10.1371/journal.pone.0268625 (PMC9200169; doi:10.1371/journal.pone.0268625)
Supplement: S2 File — This supplement includes forest plots and additional models. (DOCX) [file pone.0268625.s002.docx]

# S3 Analyses

| Section | Content | Page |
| --- | --- | --- |
| I | Outcome assessment: forest plots (Figures S1 through S9) | 2 |
| II | Additional models (Tables S3 through S10) | 8 |
| III | Summary of findings (Tables S11 and S12) | 24 |
| IV | References | 25 |

# Section II. Outcome assessment: forest plots

Data was unavailable from two studies. Group level data could not be meaningfully interpreted in regard to our research question around potential acute-chronic exercise interaction on antibody response.

Available shared data is thus presented.

Forest plots are shown by strain and outcome measurement, examining the effect of acute exercise compared to rested control on vaccine immunogenicity.

Study numbers correspond to those in the full manuscript.

Due to low numbers of physically inactive participants in each study, the data does lend not itself to meaningful analysis of a study-level effect of physical activity or of the acute exercise-physical activity interaction on vaccine immunogenicity.

The forest plots indicate little heterogeneity (I^2^ = 0-13%) between studies for seroconversion and seroprotection. Heterogeneity on the continuous measures was low-to-moderate (I^2^ = 0-35%).

##
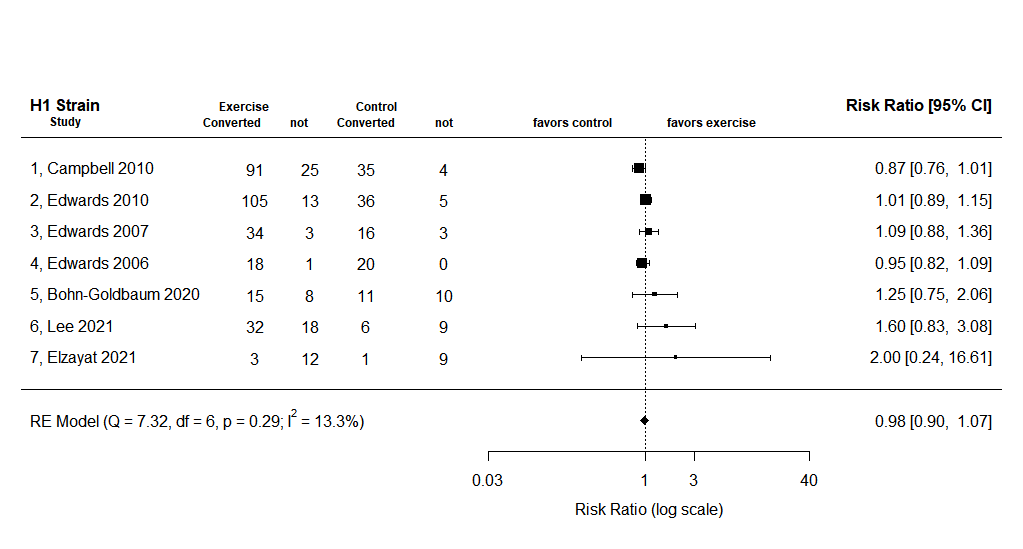
**Figure S1. Forest plot of H1 strain seroconversion**

Figure S2. Forest plot of H3 strain seroconversion


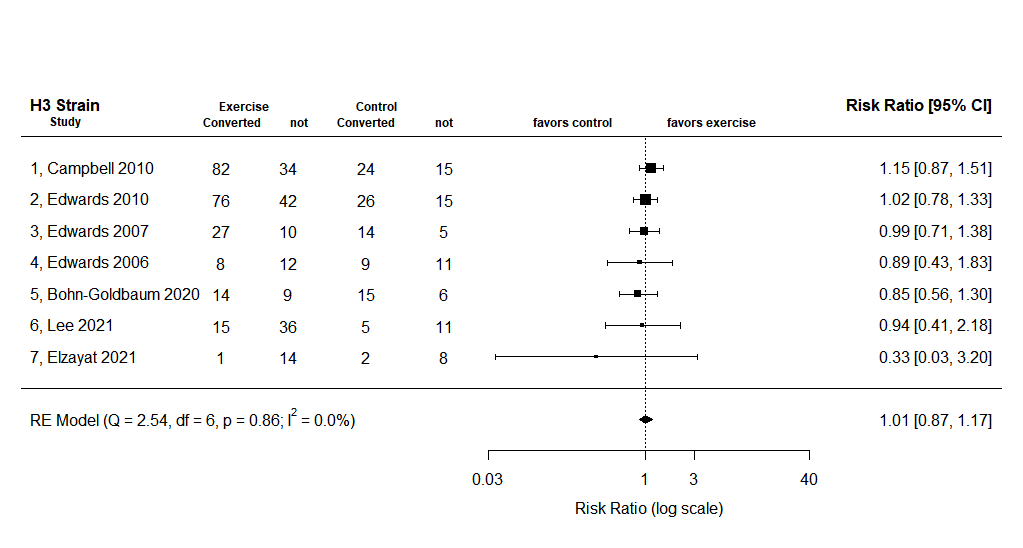


Figure S3. Forest plot of B strain seroconversion


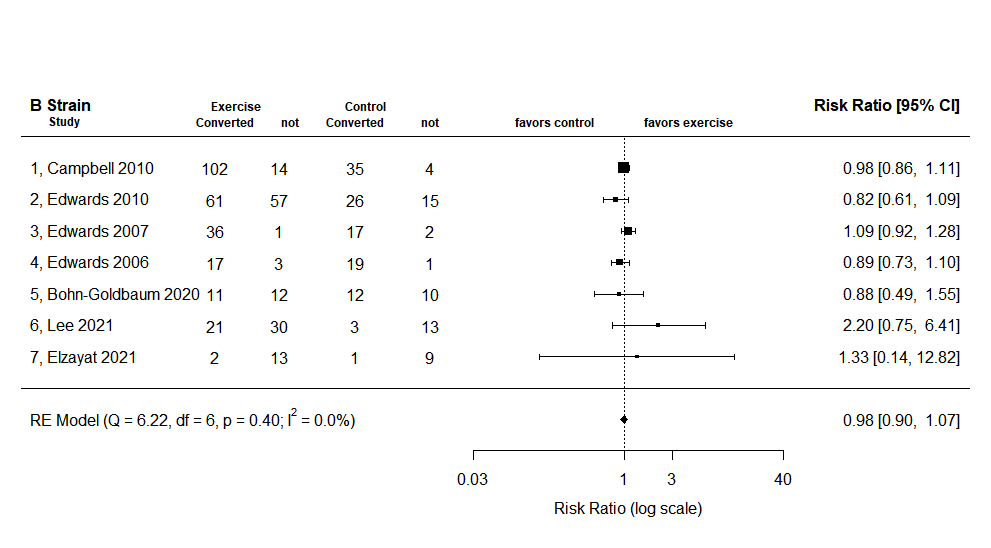


## **Figure S4. Forest plot of H1 strain seroprotection**


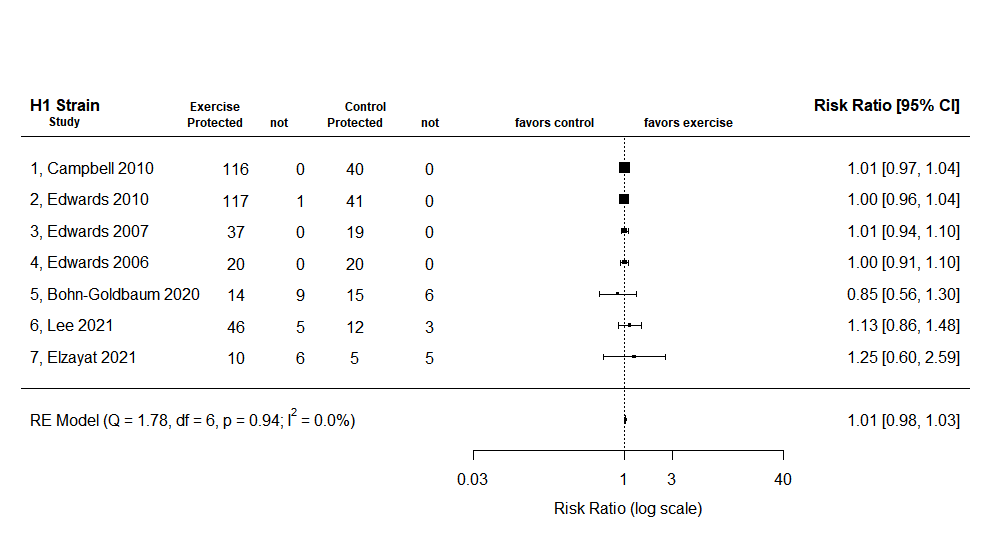


## **Figure S5. Forest plot of H3 strain seroprotection**


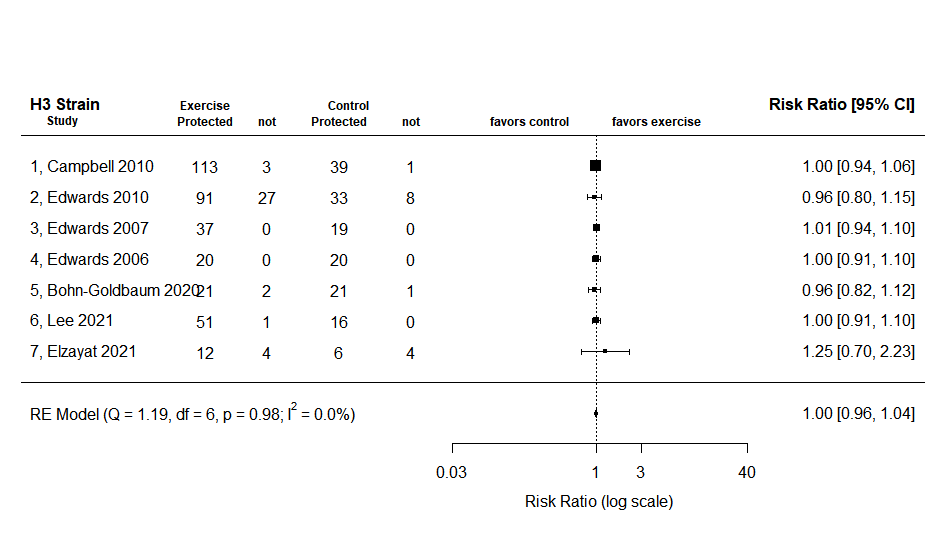


## **Figure S6. Forest plot of B strain seroprotection**


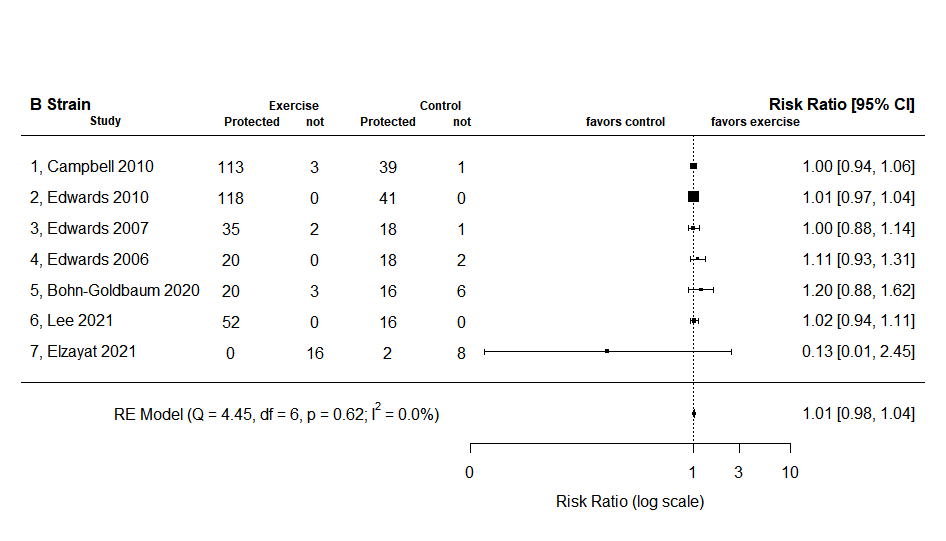


## **Figure S7. Forest plot of H1 strain titer change**


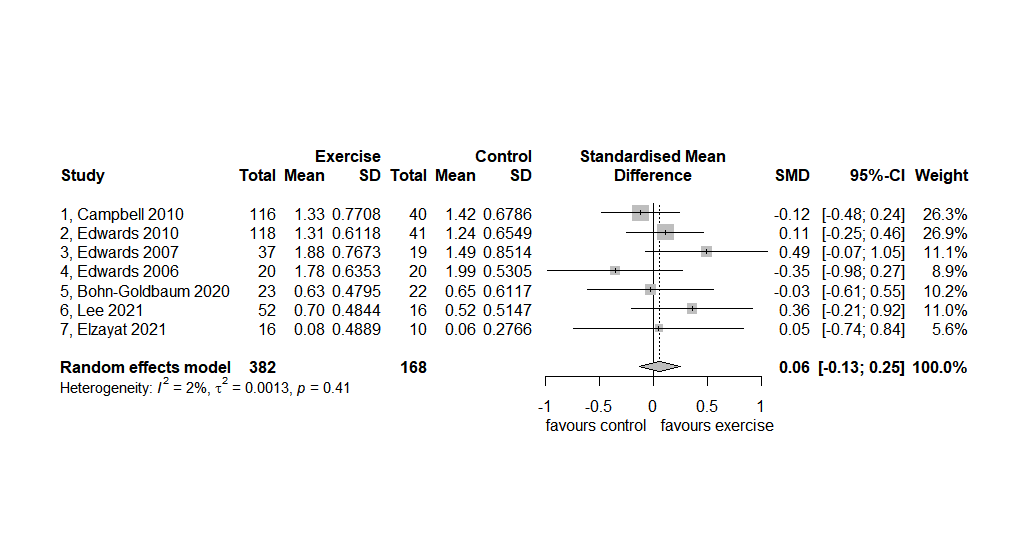


## **Figure S8. Forest plot of H3 strain titer change**


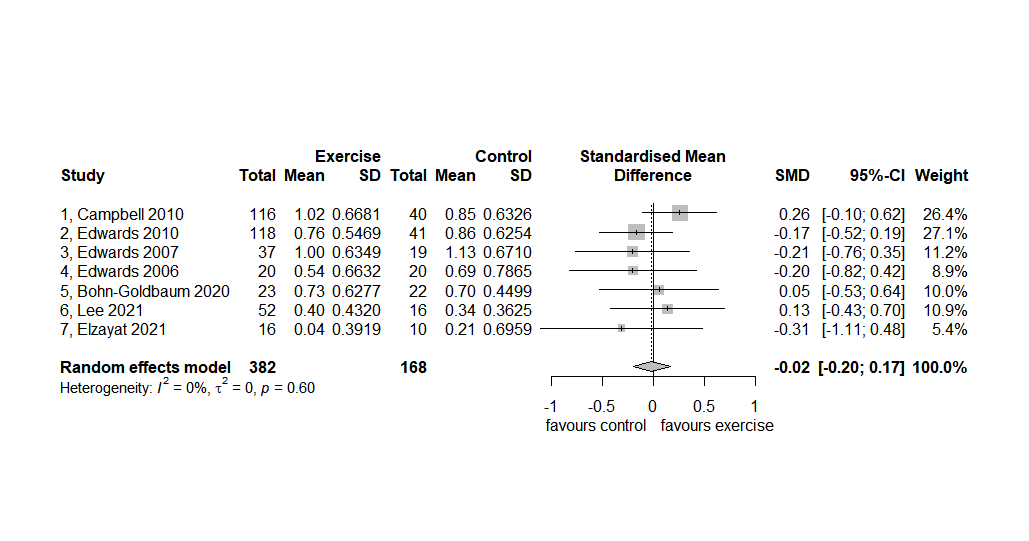


## **Figure S9. Forest plot of B strain titer change**


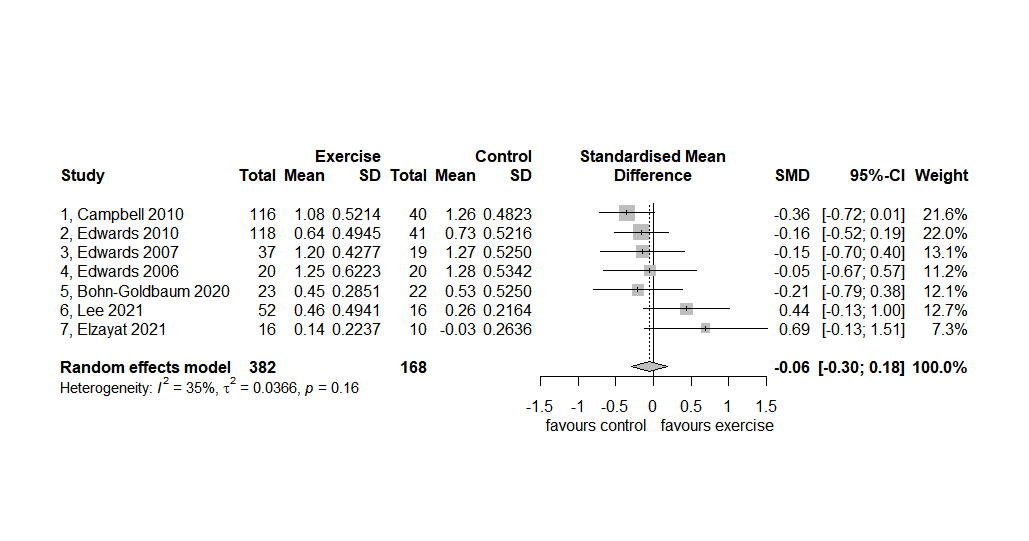


# Section IV. Additional models

## Table S3. Model 1: The effect of acute exercise on titer change from baseline to follow-up (full results).

|  | **population** | **All participants** | | | | **Physically active participants** | | | | **Inactive participants** | | | |
| --- | --- | --- | --- | --- | --- | --- | --- | --- | --- | --- | --- | --- | --- |
|  |  | Unadjusted model | | Adjusted model | | Unadjusted model | | Adjusted model | | Unadjusted model | | Adjusted model | |
| **strain** | **Predictors** | **OR (95%CI)** | **p** | **OR (95%CI)** | **p** | **OR (95%CI)** | **p** | **OR (95%CI)** | **p** | **OR (95%CI)** | **p** | **OR (95%CI)** | **p** |
| H1 | Exercise (control) | 1.01 (0.919-1.105) | 0.55 | 1.04 (0.922-1.178) | 0.50 | 1.14 (0.974-1.327) | 0.10 **^b^** | 1.14 (0.975-1.331) | 0.10 **^b^** | 0.90 (0.721-1.120) | 0.341 | 0.89 (0.705-1.098) | 0.30 |
|  | Age (year) |  |  | 0.98 (0.973-0.994) | <0.01^b^ |  |  | 0.98 (0.973-0.997) | 0.02^b^ |  |  | 0.98 (0.968-0.989) | <0.01^b^ |
|  | Sex (male) |  |  | 1.04 (0.933-1.165) | 0.46 |  |  | 1.054 (0.915-1.216) | 0.47 |  |  | 1.06 (0.875-1.277) | 0.55 |
|  | BMI (kg/m^2^) |  |  | 1.00 (0.986-1.022) | 0.67 |  |  | 1.01 (0.982-1.033) | 0.57 |  |  | 1.01 (0.983-1.038) | 0.52 |
|  | Random Effects |  |  |  |  |  |  |  |  |  |  |  |  |
|  | I^2^ | 0.29 |  | 0.08 |  | 0.29 |  | 0.09 |  | 0.27 |  | 0.03 |  |
|  | Observations | 542 |  | 539 |  | 333 |  | 331 |  | 197 |  | 196 |  |
| H3 | Exercise (control) | 0.99 (0.887-1.108) | 0.88 | 0.98 (0.881-1.100) | 0.79 | 0.99 (0.863-1.132) | 0.87 | 0.97 (0.850-1.115) | 0.71 | 0.97 (0.785-1.199) | 0.77 | 0.97 (0.777-1.183) | 0.74 |
|  | Age (year) |  |  | 0.99 (0.987-1.003) | 0.20 |  |  | 1.00 (0.990-1.007) | 0.72 |  |  | 0.99 (0.980-0.999) | 0.04^b^ |
|  | Sex (male) |  |  | 1.04 (0.943-1.154) | 0.42 |  |  | 1.082 (0.956-1.227) | 0.22 |  |  | 1.01 (0.837-1.207) | 0.91 |
|  | BMI (kg/m2) |  |  | 0.98 (0.968-1.000) | 0.05^a,b^ |  |  | 0.98 (0.958-1.001) | 0.07 |  |  | 0.99 (0.963-1.016) | 0.35 |
|  | Random Effects |  |  |  |  |  |  |  |  |  |  |  |  |
|  | I^2^ | 0.03 |  | 0.02 |  | 0.02 |  | 0.01 |  | 0.03 |  | 0.02 |  |
|  | Observations | 545 |  | 542 |  | 336 |  | 334 |  | 197 |  | 196 |  |
| B | Exercise (control) | 0.94 (0.861-1.031) | 0.2 | 0.94 (0.862-1.033) | 0.22 | 0.94 (0.842-1.058) | 0.33 | 0.95 (0.848-1.064) | 0.39 | 0.92 (0.777-1.084) | 0.31 | 0.91 (0.769-1.072) | 0.28 |
|  | Age (year) |  |  | 0.99 (0.980-0.996) | <0.01 ^b^ |  |  | 0.99 (0.979-0.996) | <0.01 ^b^ |  |  | 0.99 (0.979-0.995) | <0.01 ^b^ |
|  | Sex (male) |  |  | 0.95 (0.876-1.032) | 0.23 |  |  | 0.96 (0.868-1.069) | 0.48 |  |  | 0.92 (0.798-1.063) | 0.28 |
|  | BMI (kg/m2) |  |  | 1.01 (0.994-1.021) | 0.28 |  |  | 1.01 (0.995-1.033) | 0.15 |  |  | 1.01 (0.986-1.028) | 0.54 |
|  | Random Effects |  |  |  |  |  |  |  |  |  |  |  |  |
|  | I^2^ | 0.14 |  | 0.05 |  | 0.11 |  | 0.06 |  | 0.16 |  | 0.01 |  |
|  | Observations | 546 |  | 543 |  | 336 |  | 334 |  | 198 |  | 197 |  |

Table S3. The effect of acute exercise on influenza antibody titer change by strain and population (physically active behavior)-showing the results of linear regression unadjusted and adjusted modelling on change in antibody titer levels. Estimates are given with 95%CI in parentheses. Predictors are by unit increase (Age and BMI) or versus comparator (Exercise and Sex). BMI = body mass index. **^a^**significant in sensitivity analysis with younger population studies (those with <65 years-old). **^b^**significant in same-arm analysis

## Table S4. Model 1-young: The effect of acute exercise on titer change from baseline to follow-up among participants <36 years of age.

|  | **population** | **All participants** | | | | | **Physically active participants** | | | | | **Inactive participants** | | | | | |  |
| --- | --- | --- | --- | --- | --- | --- | --- | --- | --- | --- | --- | --- | --- | --- | --- | --- | --- | --- |
|  |  | Unadjusted model | | | Adjusted model | | Unadjusted model | | Adjusted model | | | Unadjusted model | | | Adjusted model | | |  |
| **strain** | **Predictors** | **Odds Ratio** | **p** | **Odds Ratio** | | **p** | **Odds Ratio** | **p** | | **Odds Ratio** | **p** | | **Odds Ratio** | **p** | | **Odds Ratio** | **p** | |
| H1 | Exercise (control) | 1.04 (0.910-1.195) | 0.538 | 1.04 (0.910-1.196) | | 0.536 | 1.15 (0.969-1.357) | 0.107 | | 1.15 (0.972-1.361) | 0.102 | | 0.87(0.654-1.114) | 0.272 | | 0.86 (0.649-1.109) | 0.265 | |
|  | Age (year) |  |  | 0.99 (0.968-1.013) | | 0.410 |  |  | | 0.99 (0.962-1.015) | 0.399 | |  |  | | 1.00 (0.950-1.04) | 0.843 | |
|  | Sex (male) |  |  | 1.04 (0.925-1.181) | | 0.481 |  |  | | 1.05 (0.901-1.227) | 0.531 | |  |  | | 1.09 (0.880-1.347) | 0.443 | |
|  | BMI (kg/m^2^) |  |  | 1.01 (0.988-1.032) | | 0.383 |  |  | | 1.01 (0.984-1.043) | 0.381 | |  |  | | 1.01 (0.978-1.049) | 0.513 | |
|  | Random Effects |  |  |  | |  |  |  | |  |  | |  |  | |  |  | |
|  | I^2^ | 0.11 |  | 0.10 | |  | 0.10 |  | | 0.09 |  | | 0.03 |  | | 0.03 |  | |
|  | Observations | 474 |  | 472 | |  | 296 |  | | 295 |  | | 167 |  | | 166 |  | |
| H3 | Exercise (control) | 0.994 (0.881-1.125) | 0.935 | 0.99 (0.876-1.118) | | 0.868 | 0.98 (0.846-1.131) | 0.761 | | 0.97 (0.838-1.116) | 0.644 | | 0.99 (0.770-1.244) | 0.912 | | 1.00 (0.776-1.252) | 0.979 | |
|  | Age (year) |  |  | 1.00 (0.983-1.024) | | 0.725 |  |  | | 1.02 (0.994-1.042) | 0.12 | |  |  | | 0.97 (0.925-1.011) | 0.176 | |
|  | Sex (male) |  |  | 1.07 (0.959-1.193) | | 0.232 |  |  | | 1.11 (0.973-1.269) | 0.124 | |  |  | | 1.01 (0.823-1.234) | 0.9 | |
|  | BMI (kg/m2) |  |  | 0.98 (0.962-1.000) | | **0.049** |  |  | | 0.98 (0.955-1.004) | 0.096 | |  |  | | 0.98 (0.954-1.019) | 0.355 | |
|  | Random Effects |  |  |  | |  |  |  | |  |  | |  |  | |  |  | |
|  | I^2^ | 0.01 |  | 0.01 | |  | 0.01 |  | | 0.02 |  | | 0.00 |  | | 0.00 |  | |
|  | Observations | 477 |  | 475 | |  | 299 |  | | 298 |  | | 167 |  | | 166 |  | |
| B | Exercise (control) | 0.93 (0.840-1.029) | 0.159 | 0.93 (0.841-1.029) | | 0.164 | 0.96 (0.851-1.089) | 0.548 | | 0.97 (0.854-1.091) | 0.572 | | 0.85 (0.691, 1.027) | 0.096 | | 0.84 (0.685-1.018) | 0.082 | |
|  | Age (year) |  |  | 0.99 (0.975-1.009) | | 0.313 |  |  | | 0.99 (0.965-1.003) | 0.11 | |  |  | | 1.01 (0.971-1.043) | 0.720 | |
|  | Sex (male) |  |  | 0.95 (0.873-1.045) | | 0.315 |  |  | | 0.96 (0.862-1.080) | 0.526 | |  |  | | 0.95 (0.803-1.114) | 0.516 | |
|  | BMI (kg/m2) |  |  | 1.01 (0.997-1.030) | | 0.109 |  |  | | 1.01 (0.994-1.036) | 0.178 | |  |  | | 1.02 (0.989-1.043) | 0.275 | |
|  | Random Effects |  |  |  | |  |  |  | |  |  | |  |  | |  |  | |
|  | I^2^ | 0.06 |  | 0.06 | |  | 0.08 |  | | 0.07 |  | | 0.01 |  | | 0.01 |  | |
|  | Observations | 477 |  | 475 | |  | 299 |  | | 298 |  | | 167 |  | | 166 |  | |

Table S4. The effect of acute exercise on influenza titer change among participants <36 years, by strain and population (physical activity level). Estimates are given with 95%CI in parentheses. Predictors are by unit increase (Age and BMI) or versus dichotomous comparator (Exercise and Sex). BMI = body mass index.

## Table S5. Model 1- same-arm: The effect of acute exercise on titer change in participants engaging in an acute exercise intervention involving the vaccinated arm.

|  | **population** | **All participants** | | | | | | **Physically active participants** | | | | **Inactive participants** | | | |
| --- | --- | --- | --- | --- | --- | --- | --- | --- | --- | --- | --- | --- | --- | --- | --- |
|  |  | Unadjusted model | | | Adjusted model | | | Unadjusted model | | Adjusted model | | Unadjusted model | | Adjusted model | |
| **strain** | **Predictors** | **Odds Ratio** | **p** | **Odds Ratio** | | **p** | **Odds Ratio** | | **p** | **Odds Ratio** | **p** | **Odds Ratio** | **p** | **Odds Ratio** | **p** |
| H1 | Exercise (control) | 1.06 (0. 9-1.207) | 0.387 | 1.06 (0.931-1.210) | | 0.354 | 1.19 (1.002-1.399) | | **0.044** | 1.19 (1.003-1.404) | **0.041** | 0.89 (0.699-1.119) | 0.310 | 0.86 (0.656-1.019) | 0.192 |
|  | Age (year) |  |  | 0.98 (0.970-0.993) | | **0.001** |  | |  | 0.98 (0.970-0.996) | **0.012** |  |  | 0.98 (0.975-0.986) | **<0.001** |
|  | Sex (male) |  |  | 1.04 (0.923-1.167) | | 0.543 |  | |  | 1.04 (0.893-1.214) | 0.614 |  |  | 1.08 (0.877-1.296) | 0.465 |
|  | BMI (kg/m^2^) |  |  | 1.00 (0.986-1.024) | | 0.636 |  | |  | 1.01 (0.980-1.035) | 0.621 |  |  | 1.01 (0.988-1.045) | 0.393 |
|  | Random Effects |  |  |  | |  |  | |  |  |  |  |  |  |  |
|  | I^2^ | 0.32 |  | 0.10 | |  | 0.35 | |  | 0.12 |  | 0.24 |  | 0.01 |  |
|  | Observations | 499 |  | 495 | |  | 297 | |  | 295 |  | 190 |  | 188 |  |
| H3 | Exercise (control) | 1.00 (0.892-1.130) | 0.956 | 1.00 (0.889-1.125) | | 0.987 | 1.00 (0.870-1.157) | | 0.997 | 0.99 (0.858-1.146) | 0.889 | 0.98 (0.786-1.221) | 0.834 | 0.98 (0.784-1.206) | 0.828 |
|  | Age (year) |  |  | 0.99 (0.985-1.001) | | 0.119 |  | |  | 1.00 (0.989-1.006) | 0.621 |  |  | 0.99 (0.979-0.999) | **0.047** |
|  | Sex (male) |  |  | 1.05 (0.941-1.163) | | 0.411 |  | |  | 1.08 (0.951-1.244) | 0.237 |  |  | 1.02 (0.837-1.217) | 0.851 |
|  | BMI (kg/m2) |  |  | 0.98 (0.966-0.999) | | **0.036** |  | |  | 0.978 (0.955-1.002) | 0.066 |  |  | 0.98 (0.956-1.013) | 0.249 |
|  | Random Effects |  |  |  | |  |  | |  |  |  |  |  |  |  |
|  | I^2^ | 0.02 |  | 0.02 | |  | 0.01 | |  | 0.01 |  | 0.03 |  | 0.02 |  |
|  | Observations | 500 |  | 496 | |  | 298 | |  | 296 |  | 190 |  | 188 |  |
| B | Exercise (control) | 0.93 (0.846-1.022) | 0.133 | 0.937 (0.847-1.024) | | 0.145 | 0.93 (0.824-1.047) | | 0.235 | 0.94 (0.8299-1.054) | 0.283 | 0.90 (0.756-1.066) | 0.219 | 0.89 (0.747-1.045 | 0.185 |
|  | Age (year) |  |  | 0.99 (0.981-0.997) | | **0.011** |  | |  | 0.99 (0.979-0.998) | **0.02** |  |  | 0.99 (0.979-0.996) | **0.002** |
|  | Sex (male) |  |  | 0.94 (0.866-1.026) | | 0.171 |  | |  | 0.95 (0.848-1.058) | 0.328 |  |  | 0.92 (0.794-1.059) | 0.247 |
|  | BMI (kg/m2) |  |  | 1.01 (0.995-1.022) | | 0.242 |  | |  | 1.01 (0.993-1.033) | 0.206 |  |  | 1.01 (0.989-1.031) | 0.384 |
|  | Random Effects |  |  |  | |  |  | |  |  |  |  |  |  |  |
|  | I^2^ | 0.15 |  | 0.06 | |  | 0.10 | |  | 0.06 |  | 0.16 |  | 0.01 |  |
|  | Observations | 501 |  | 497 | |  | 298 | |  | 296 |  | 191 |  | 189 |  |

Table S5. The effect of acute exercise on influenza titer change among participants engaging in an acute exercise intervention involving the vaccinated arm, by strain and population (physical activity level). Estimates are given with 95%CI in parentheses. Predictors are by unit increase (Age and BMI) or versus dichotomous comparator (Exercise and Sex). BMI = body mass index.

## Table S6. Model 2: The effect of physical activity level on titer change from baseline to follow-up (full results).

|  | **population** | **All participants** | | | | | | | | | | | **Exercised participants** | | | | | | | | | | | **Control participants** | | | | | | | |  |
| --- | --- | --- | --- | --- | --- | --- | --- | --- | --- | --- | --- | --- | --- | --- | --- | --- | --- | --- | --- | --- | --- | --- | --- | --- | --- | --- | --- | --- | --- | --- | --- | --- |
|  |  | Unadjusted model | | | | | | Adjusted model | | | | | Unadjusted model | | | | | Adjusted model | | | | | Unadjusted model | | | | | | Adjusted model | | |  |
| **strain** | **Predictors** | **OR (95%CI)** | | **p** | | **OR (95%CI)** | | | **p** | | **OR (95%CI)** | | | **p** | | **OR (95%CI)** | | | **p** | | **OR (95%CI)** | | | | **p** | | **OR (95%CI)** | | | **p** | | |
| H1 | PA level (inactive) | | 1.12 (0.993-1.27) | | 0.06 **^b^** | | 1.12 (0.991-1.269) | | | 0.07 **^b^** | | 1.21 (1.039-1.404) | | | 0.01 ^a,b^ | | 1.20 (1.034-1.398) | | | 0.02 ^a,b^ | | 0.96 (0.766-1.206) | | | | 0.71 | | 0.95 (0.756-1.184) | | | 0.63 | |
|  | Age (year) | |  | |  | | 0.98 (0.973- 0.994) | | | <0.01^b^ | |  | | |  | | .99 (0.974-0.997) | | | 0.01^b^ | |  | | | |  | | 0.98 (0.965-0.992) | | | <0.01^b^ | |
|  | Sex (male) | |  | |  | | 1.05 (0.938-1.174) | | | 0.41 | |  | | |  | | 1.07 (0.939-1.230) | | | 0.30 | |  | | | |  | | 1.01 (0.822-1.235) | | | 0.94 | |
|  | BMI (kg/m^2^) | |  | |  | | 1.00 (0.987-1.023) | | | 0.60 | |  | | |  | | 1.01 (0.983-1.028) | | | 0.65 | |  | | | |  | | 1.01 (0.975-1.041) | | | 0.67 | |
|  | Random Effects | |  | |  | |  | | |  | |  | | |  | |  | | |  | |  | | | |  | |  | | |  | |
|  | I^2^ | | 0.28 | |  | | 0.08 | | |  | | 0.27 | | |  | | 0.07 | | |  | | 0.30 | | | |  | | 0.30 | | |  | |
|  | Observations | | 530 | |  | | 527 | | |  | | 372 | | |  | | 370 | | |  | | 158 | | | |  | | 157 | | |  | |
| H3 | PA level (inactive) | | 0.96 (0.861-1.077) | | 0.51 | | 0.95 (0.850-1.065) | | | 0.39 | | 0.98 (0.855-1.119\ | | | 0.75 | | 0.96 (0.838-1.097) | | | 0.55 | | 0.96 (0.775-1.193) | | | | 0.69 | | 0.94 (0.761-1.167) | | | 0.58 | |
|  | Age (year) | |  | |  | | 0.99 (0.987-1.003) | | | 0.21 | |  | | |  | | 0.99 (0.986-1.003) | | | 0.22 | |  | | | |  | | 0.99 (0.985-1.003) | | | 0.24 | |
|  | Sex (male) | |  | |  | | 1.05 (0.948-1.163) | | | 0.35 | |  | | |  | | 1.01 (0.892-1.134) | | | 0.93 | |  | | | |  | | 1.16 (0.956-1.413) | | | 0.13 | |
|  | BMI (kg/m2) | |  | |  | | 0.98 (0.967-1.000) | | | <0.05^b^ | |  | | |  | | 0.98 (0.964-1.003) | | | 0.09 | |  | | | |  | | 0.99 (0.958-1.020) | | | 0.43 | |
|  | Random Effects | |  | |  | |  | | |  | |  | | |  | |  | | |  | |  | | | |  | |  | | |  | |
|  | I^2^ | | 0.03 | |  | | 0.02 | | |  | | 0.03 | | |  | | 0.02 | | |  | | 0.01 | | | |  | | 0.01 | | |  | |
|  | Observations | | 533 | |  | | 530 | | |  | | 374 | | |  | | 372 | | |  | | 159 | | | |  | | 158 | | |  | |
| B | PA level (inactive) | | 1.01 (0.921-1.108) | | 0.84 | | 1.01 (0.919-1.105) | | | 0.87 | | 0.99 (0.890-1.114) | | | 0.94 | | 0.99 (0.888-1.111) | | | 0.89 | | 1.01 (0.855-1.204) | | | | 0.89 | | 1.01 (0.849-1.192) | | | 0.95 | |
|  | Age (year) | |  | |  | | 0.99 (0.980-0.996) | | | <0.01 ^b^ | |  | | |  | | 0.99 (0.981-0.998) | | | 0.01 | |  | | | |  | | 0.99 (0.974-0.997) | | | 0.02^b^ | |
|  | Sex (male) | |  | |  | | 0.95 (0.873-1.032) | | | 0.22 | |  | | |  | | 0.94 (0.847-1.034) | | | 0.19 | |  | | | |  | | 0.99 (0.846-1.150) | | | 0.86 | |
|  | BMI (kg/m2) | |  | |  | | 1.01 (0.995-1.023) | | | 0.20 | |  | | |  | | 1.01 (0.994-1.028) | | | 0.20 | |  | | | |  | | 1.00 (0.978-1.027) | | | 0.88 | |
|  | Random Effects | |  | |  | |  | | |  | |  | | |  | |  | | |  | |  | | | |  | |  | | |  | |
|  | I^2^ | | 0.14 | |  | | 0.05 | | |  | | 0.11 | | |  | | 0.04 | | |  | | 0.22 | | | |  | | 0.11 | | |  | |
|  | Observations | | 534 | |  | | 531 | | |  | | 374 | | |  | | 372 | | |  | | 160 | | | |  | | 159 | | |  | |

Table S6. The effect of physical activity (PA) level on influenza antibody titer change by strain and population (intervention)-showing the results of linear regression unadjusted and adjusted modelling on change in antibody titer levels. Predictors are by unit increase (Age and BMI) or versus dichotomous comparator (PA level and Sex). Estimates are given with 95%CI in parentheses. BMI = body mass index. **^a^**significant in sensitivity analysis with younger population studies (those with <36 years-old). **^b^**significant in same-arm analysis

## Table S7. Model 2-young: The effect of physical activity level on titer change from baseline to follow-up among participants <36 years of age.

|  | **population** | **All participants** | | | | | | **Exercised participants** | | | | **Control participants** | | | |
| --- | --- | --- | --- | --- | --- | --- | --- | --- | --- | --- | --- | --- | --- | --- | --- |
|  |  | Unadjusted model | | | Adjusted model | | | Unadjusted model | | Adjusted model | | Unadjusted model | | Adjusted model | |
| **strain** | **Predictors** | **Odds Ratio** | **p** | **Odds Ratio** | | **p** | **Odds Ratio** | | **p** | **Odds Ratio** | **p** | **Odds Ratio** | **p** | **Odds Ratio** | **p** |
| H1 | PA level (inactive) | 1.11 (0.970-1.278) | 0.126 | 1.12 (0.972-1.281) | | 0.119 | 1.20 (1.017-1.411) | | **0.031** | 1.20 (1.021-1.417) | **0.028** | 0.91 (0.700-1.182) | 0.489 | 0.92 (0.708-1.191) | 0.536 |
|  | Age (year) |  |  | 0.99 (0.965-1.011) | | 0.307 |  | |  | 1.00 (0.971-1.026) | 0.909 |  |  | 0.97 (0.929-1.012) | 0.166 |
|  | Sex (male) |  |  | 1.05 (0.930-1.191) | | 0.421 |  | |  | 1.07 (0.929-1.243) | 0.337 |  |  | 1.02 (0.809-1.289) | 0.864 |
|  | BMI (kg/m^2^) |  |  | 1.01 (0.987-1.032) | | 0.415 |  | |  | 1.01 (0.987-1.040) | 0.349 |  |  | 1.01 (0.967-1.048) | 0.775 |
|  | Random Effects |  |  |  | |  |  | |  |  |  |  |  |  |  |
|  | I^2^ | 0.10 |  | 0.09 | |  | 0.10 | |  | 0.08 |  | 0.12 |  | 0.11 |  |
|  | Observations | 463 |  | 461 | |  | 335 | |  | 333 |  | 128 |  | 128 |  |
| H3 | PA level (inactive) | 0.95 (0.839-1.073) | 0.408 | 0.94 (0.831-1.063) | | 0.328 | 0.95 (0.823-1.097) | | 0.493 | 0.94 (0.811-1.082) | 0.382 | 0.94 (0.725-1.194) | 0.595 | 0.95 (0.738-1.202) | 0.653 |
|  | Age (year) |  |  | 1.01 (0.985-1.027) | | 0.572 |  | |  | 1.00 (0.977-1.027) | 0.862 |  |  | 1.00 (0.961-1.042) | 0.893 |
|  | Sex (male) |  |  | 1.08 (0.966-1.204) | | 0.183 |  | |  | 1.03 (0.907-1.171) | 0.657 |  |  | 1.22 (0.9832253-1.522377) | 0.077 |
|  | BMI (kg/m2) |  |  | 0.98 (0.962-1.001) | | 0.058 |  | |  | 0.99 (0.964-1.009) | 0.242 |  |  | 0.97 (0.935-1.009) | 0.250 |
|  | Random Effects |  |  |  | |  |  | |  |  |  |  |  |  |  |
|  | I^2^ | 0.01 |  | 0.01 | |  | 0.01 | |  | 0.01 |  | 0.01 |  | 0.01 |  |
|  | Observations | 466 |  | 464 | |  | 337 | |  | 335 |  | 129 |  | 129 |  |
| B | PA level (inactive) | 0.98 (0.880-1.081) | 0.632 | 0.97 (0.879-1.080) | | 0.620 | 0.99 (0.872-1.116) | | 0.819 | 0.99 (0.871-1.115) | 0.810 | 0.92 (0.756-1.112) | 0.384 | 0.92 (0.758-1.114) | 0.400 |
|  | Age (year) |  |  | 0.99 (0.972-1.006) | | 0.203 |  | |  | 0.99 (0.970-1.012) | 0.389 |  |  | 0.99 (0.956-1.019) | 0.432 |
|  | Sex (male) |  |  | 0.95 (0.870-1.046) | | 0.315 |  | |  | 0.95 (0.850-1.057) | 0.334 |  |  | 0.97 (0.821-1.158) | 0.769 |
|  | BMI (kg/m2) |  |  | 1.01 (0.997-1.030) | | 0.108 |  | |  | 1.02 (0.997-1.037) | 0.097 |  |  | 1.00 (0.975-1.036) | 0.760 |
|  | Random Effects |  |  |  | |  |  | |  |  |  |  |  |  |  |
|  | I^2^ | 0.07 |  | 0.06 | |  | 0.05 | |  | 0.05 |  | 0.12 |  | 0.10 |  |
|  | Observations | 466 |  | 464 | |  | 337 | |  | 335 |  | 129 |  | 129 |  |

Table S7. The effect of physical activity (PA) on influenza titer change among participants <36years, by strain and population (intervention). Estimates are given with 95%CI in parentheses. BMI = body mass index. Predictors are by unit increase (Age and BMI) or versus dichotomous comparator (PA level and Sex).

## Table S8. Model 2-same arm: The effect of physical activity on titer change in participants engaging in an acute exercise intervention involving the vaccinated arm.

|  | **population** | **All participants** | | | | | | **Exercise participants** | | | | **Control participants** | | | |
| --- | --- | --- | --- | --- | --- | --- | --- | --- | --- | --- | --- | --- | --- | --- | --- |
|  |  | Unadjusted model | | | Adjusted model | | | Unadjusted model | | Adjusted model | | Unadjusted model | | Adjusted model | |
| **strain** | **Predictors** | **Odds Ratio** | **p** | **Odds Ratio** | | **p** | **Odds Ratio** | | **p** | **Odds Ratio** | **p** | **Odds Ratio** | **p** | **Odds Ratio** | **p** |
| H1 | PA level (inactive) | 1.15 (1.016-1.314) | **0.028** | 1.16 (1.017-1.318) | | **0.027** | 1.26 (1.073-1.476) | | **0.005** | 1.27 (1.078-1.485) | **0.004** | 0.96 (0.766-1.206) | 0.71 | 0.95 (0.756-1.184) | 0.634 |
|  | Age (year) |  |  | 0.98 (0.971-0.993) | | **0.002** |  | |  | 0.99 (0.974-0.997) | **0.019** |  |  | 0.98 (0.965-0.992) | **0.003** |
|  | Sex (male) |  |  | 1.05 (0.928-1.178) | | 0.468 |  | |  | 1.08 (0.932-1.250) | 0.318 |  |  | 1.01 (0.822-1.235) | 0.94 |
|  | BMI (kg/m^2^) |  |  | 1.01 (0.987-1.025) | | 0.56 |  | |  | 1.01 (0.983-1.032) | 0.575 |  |  | 1.01 (0.975-1.041) | 0.672 |
|  | Random Effects |  |  |  | |  |  | |  |  |  |  |  |  |  |
|  | I^2^ | 0.29 |  | 0.09 | |  | 0.23 | |  | 0.06 |  | 0.30 |  | 0.10 |  |
|  | Observations | 487 |  | 483 | |  | 329 | |  | 326 |  | 158 |  | 157 |  |
| H3 | PA level (inactive) | 0.98 (0.869-1.097) | 0.675 | 0.96 (0.857-1.082) | | 0.521 | 0.99 (0.863-1.145) | | 0.938 | 0.97 (0.843-1.118) | 0.686 | 0.96 (0.775-1.193) | 0.694 | 0.94 (0.761-1.167) | 0.579 |
|  | Age (year) |  |  | 0.99 (0.985-1.001) | | 0.12 |  | |  | 0.99 (0.982-1.002) | 0.128 |  |  | 0.99 (0.985-1.003) | 0.236 |
|  | Sex (male) |  |  | 1.05 (0.947-1.174) | | 0.339 |  | |  | 1.00 (0.884-1.146) | 0.949 |  |  | 1.162 (0.956-1.413) | 0.132 |
|  | BMI (kg/m2) |  |  | 0.98 (0.965-0.999) | | **0.038** |  | |  | 0.98 (0.960-1.002) | 0.076 |  |  | 0.99 (0.958-1.020) | 0.426 |
|  | Random Effects |  |  |  | |  |  | |  |  |  |  |  |  |  |
|  | I^2^ | 0.03 |  | 0.02 | |  | 0.04 | |  | 0.03 |  | 0.01 |  | 0.01 |  |
|  | Observations | 488 |  | 484 | |  | 329 | |  | 326 |  | 159 |  | 158 |  |
| B | PA level (inactive) | 1.03 (0.937-1.130) | 0.559 | 1.03 (0.934-1.127) | | 0.598 | 1.02 (0.910-1.143) | | 0.745 | 1.02 (0.907-1.141) | 0.778 | 1.01 (0.855-1.204) | 0.888 | 1.01 (0.849-1.192) | 0.950 |
|  | Age (year) |  |  | 0.99 (0.981-0.997) | | 0.011 |  | |  | 0.99 (0.983-1.000) | 0.064 |  |  | 0.99 (0.974-0.997) | 0.016 |
|  | Sex (male) |  |  | 0.94 (0.864-1.027) | | 0.172 |  | |  | 0.92 (0.833-1.028) | 0.142 |  |  | 0.99 (0.846-1.150) | 0.859 |
|  | BMI (kg/m2) |  |  | 1.01 (0.996-1.025) | | 0.154 |  | |  | 1.01 (0.996-1.031) | 0.138 |  |  | 1.00 (0.978-1.027) | 0.884 |
|  | Random Effects |  |  |  | |  |  | |  |  |  |  |  |  |  |
|  | I^2^ | 0.15 |  | 0.06 | |  | 0.08 | |  | 0.03 |  | 0.22 |  | 0.11 |  |
|  | Observations | 489 |  | 485 | |  | 329 | |  | 326 |  | 160 |  | 159 |  |

Table S8. The effect of physical activity on influenza titer change among participants engaging in an acute exercise intervention involving the vaccinated arm, by strain and population (physical activity level). Estimates are given with 95%CI in parentheses. Predictors are by unit increase (Age and BMI) or versus dichotomous comparator (Exercise and Sex). BMI = body mass index.

## Table S9. Model 3: The effect of acute exercise on seroconversion

|  | population | All participants | | | | | Physically active participants | | | | | Inactive participants | | | | |
| --- | --- | --- | --- | --- | --- | --- | --- | --- | --- | --- | --- | --- | --- | --- | --- | --- |
|  |  | Unadjusted model | | Adjusted model | | | Unadjusted model | | Adjusted model | | | Unadjusted model | | Adjusted model | | |
| strain | Predictors | Odds Ratio | p | | Odds Ratio | p | Odds Ratio | p | | Odds Ratio | p | Odds Ratio | p | | Odds Ratio | p |
| H1 | Exercise (control) | 1.15 (0.701- 1.876) | 0.573 | | 1.12 (0.678-1.830) | 0.649 | 1.35 (0.703- 2.546) | 0.357 | | 1.25 (0.629- 2.033) | 0.498 | 0.86 (0.341- 1.992) | 0.707 | | 0.76 (0.304-1.760) | 0.534 |
|  | Age (year) |  |  | | 0.96 (0.935-0.994) | 0.007 |  |  | | 0.98 (0.946-1.007) | 0.127 |  |  | | 0.944 (0.915-0.968) | <0.001 |
|  | Sex (male) |  |  | | 0.80 (0.507-1.257) | 0.331 |  |  | | 1.00 (0.556-1.799) | 0.998 |  |  | | 0.64 (0.296-1.336) | 0.236 |
|  | BMI (kg/m2) |  |  | | 0.99 (0.927-1.061) | 0.791 |  |  | | 0.937 (0.849-1.034) | 0.194 |  |  | | 1.09 (0.979-1.223) | 0.133 |
|  | Random Effects |  |  | |  |  |  |  | |  |  |  |  | |  |  |
|  | I^2^ | 0.56 |  | | 0.15 |  | 0.46 |  | | 0.17 |  | 0.68 |  | | 0.00 |  |
|  | Observations | 543 |  | | 539 |  | 333 |  | | 331 |  | 198 |  | | 196 |  |
| H3 | Exercise (control) | 1.01 (0.677- 1.500) | 0.963 | | 0.98 (0.656-1.463) | 0.927 | 1.07 (0.643- 1.759) | 0.804 | | 1.01 (0.607-1.685) | 0.958 | 0.90 (0.438- 1.798) | 0.766 | | 0.86 (0.420-1.741) | 0.686 |
|  | Age (year) |  |  | | 0.98 (0.952-1.009) | 0.211 |  |  | | 0.99 (0.959-1.0177) | 0.423 |  |  | | 0.98 (0.939-1.013) | 0.284 |
|  | Sex (male) |  |  | | 1.08 (0.754-1.559) | 0.663 |  |  | | 1.31 (0.817-2.091) | 0.264 |  |  | | 0.94 (0.513-1.719) | 0.836 |
|  | BMI (kg/m2) |  |  | | 0.95 (0.898-1.010) | 0.101 |  |  | | 0.93 (0.852-1.007) | 0.074 |  |  | | 0.96 (0.878-1.049) | 0.372 |
|  | Random Effects |  |  | |  |  |  |  | |  |  |  |  | |  |  |
|  | I^2^ | 0.16 |  | | 0.13 |  | 0.13 |  | | 0.09 |  | 0.10 |  | | 0.07 |  |
|  | Observations | 545 |  | | 542 |  | 336 |  | | 334 |  | 197 |  | | 196 |  |
| B | Exercise (control) | 0.90 (0.568- 1.414) | 0.644 | | 0.89 (0.562-1.413) | 0.631 | 1.13 (0.629-2.033) | 0.672 | | 1.16 (0.639-2.104) | 0.619 | 0.55 (0.238- 1.214) | 0.145 | | 0.50 (0.212-1.114) | 0.097 |
|  | Age (year) |  |  | | 0.95 (0.919-0.987) | **0.006** |  |  | | 0.96 (0.921-0.997) | **0.031** |  |  | | 0.95 (0.908-0.979) | **0.002** |
|  | Sex (male) |  |  | | 0.90 (0.593-1.357) | 0.607 |  |  | | 1.35 (0.780-2.336) | 0.283 |  |  | | 0.53 (0.268-1.029) | 0.061 |
|  | BMI (kg/m2) |  |  | | 1.01 (0.950-1.081) | 0.698 |  |  | | 1.01 (0.915-1.105) | 0.911 |  |  | | 1.06 (0.963-1.172) | 0.241 |
|  | Random Effects |  |  | |  |  |  |  | |  |  |  |  | |  |  |
|  | I^2^ | 0.64 |  | | 0.38 |  | 0.54 |  | | 0.35 |  | 0.66 |  | | 0.10 |  |
|  | Observations | 546 |  | | 543 |  | 336 |  | | 334 |  | 198 |  | | 197 |  |

Table S9. The effect of acute exercise on influenza antibody conversion, by strain and population (physical activity level). Predictors are by unit increase or versus dichotomous comparator. Predictors are by unit increase (Age and BMI) or versus dichotomous comparator (Exercise and Sex). BMI = body mass index.

## S10 Table. Model 4: The effect of physical activity level on seroconversion

|  | population | All participants | | | | | | | | Exercise participants | | | | | | | | | Control participants | | | | | | | | |
| --- | --- | --- | --- | --- | --- | --- | --- | --- | --- | --- | --- | --- | --- | --- | --- | --- | --- | --- | --- | --- | --- | --- | --- | --- | --- | --- | --- |
|  |  | Unadjusted model | | | | Adjusted model | | | | Unadjusted model | | | Adjusted model | | | | | | Unadjusted model | | | Adjusted model | | | | | |
| strain | Predictors | Odds Ratio | p | Odds Ratio | | | p | | Odds Ratio | | p | | | Odds Ratio | | p | | Odds Ratio | | p | | | Odds Ratio | | p | |  |
| H1 | PA level (inactive) | 1.73 (1.047- 2.875) | **0.032** | | 1.69 (1.022-2.817) | | | **0.040** | | 1.79 (0.990-3.278) | | 0.054 | | | 1.74 (0.959-3.194) | | 0.069 | | 1.47 (0.539-3.899) | | 0.430 | | | 1.27 (0.457-3.431) | | 0.628 | |
|  | Age (year) |  |  | | 0.96 (0.936-0.997) | | | **0.012** | |  | |  | | | 0.97 (0.941-0.999) | | **0.020** | |  | |  | | | 0.95 (0.899-0.992) | | **0.017** | |
|  | Sex (male) |  |  | | 0.81 (0.510-1.279) | | | 0.362 | |  | |  | | | 0.70 (0.406-1.201) | | 0.196 | |  | |  | | | 1.32 (0.540-3.317) | | 0.535 | |
|  | BMI (kg/m2) |  |  | | 1.00 (0.929-1.068) | | | 0.892 | |  | |  | | | 1.00 (0.918-1.0836) | | 0.926 | |  | |  | | | 1.01 (0.881-1.160) | | 0.909 | |
|  | Random Effects |  |  | |  | | |  | |  | |  | | |  | |  | |  | |  | | |  | |  | |
|  | I^2^ | 0.55 |  | | 0.17 | | |  | | 0.37 | |  | | | 0.08 | |  | | 0.73 | |  | | | 0.35 | |  | |
|  | Observations | 531 |  | | 527 | | |  | | 373 | |  | | | 370 | |  | | 158 | |  | | | 157 | |  | |
| H3 | PA level (inactive) | 1.03 (0.692-1.543) | 0.840 | | 1.00 (0.666-1.496) | | | 0.994 | | 1.20 (0.727- 1.938) | | 0.496 | | | 1.11 (0.680-1.834) | | 0.668 | | 0.90 (0.435- 1.812) | | 0.767 | | | 0.84 (0.397-1.726) | | 0.633 | |
|  | Age (year) |  |  | | 0.98 (0.936-0.997) | | | 0.211 | |  | |  | | | 0.99 (0.955-1.016) | | 0.327 | |  | |  | | | 0.99 (0.950-1.014) | | 0.368 | |
|  | Sex (male) |  |  | | 1.15 (0.510-1.279) | | | 0.463 | |  | |  | | | 0.93 (0.594-1.442) | | 0.736 | |  | |  | | | 1.84 (0.948-3.621) | | 0.074 | |
|  | BMI (kg/m2) |  |  | | 0.94 (0.929-1.068) | | | 0.056 | |  | |  | | | 0.93 (0.860-0.997) | | **0.043** | |  | |  | | | 0.99 (0.891-1.107) | | 0.892 | |
|  | Random Effects |  |  | |  | | |  | |  | |  | | |  | |  | |  | |  | | |  | |  | |
|  | I^2^ | 0.16 |  | | 0.13 | | |  | | 0.19 | |  | | | 0.12 | |  | | 0.02 | |  | | | 0.03 | |  | |
|  | Observations | 534 |  | | 530 | | |  | | 375 | |  | | | 372 | |  | | 159 | |  | | | 158 | |  | |
| B | PA level (inactive) | 1.27 (0.812- 1.988) | 0.291 | | 1.25 (0.791-1.961) | | | 0.339 | | 1.376(0.800- 2.335) | | 0.251 | | | 1.33 (0.775-2.304) | | 0.296 | | 0.98 (0.406- 2.304) | | 0.968 | | | 0.88 (0.356-2.105) | | 0.778 | |
|  | Age (year) |  |  | | 0.95 (0.919-0.988) | | | **0.007** | |  | |  | | | 0.96 (0.918-0.992) | | **0.014** | |  | |  | | | 0.95 (0.900-0.998) | | **0.039** | |
|  | Sex (male) |  |  | | 0.89 (0.585-1.349) | | | 0.578 | |  | |  | | | 0.82 (0.500-1.344) | | 0.433 | |  | |  | | | 1.16 (0.520-2.607) | | 0.712 | |
|  | BMI (kg/m2) |  |  | | 1.02 (0.958-1.094) | | | 0.503 | |  | |  | | | 1.02 (0.939-1.100) | | 0.702 | |  | |  | | | 1.05 (0.929-1.205) | | 0.418 | |
|  | Random Effects |  |  | |  | | |  | |  | |  | | |  | |  | |  | |  | | |  | |  | |
|  | I^2^ | 0.62 |  | | 0.38 | | |  | | 0.61 | |  | | | 0.35 | |  | | 0.69 | |  | | | 0.48 | |  | |
|  | Observations | 535 |  | | 531 | | |  | | 375 | |  | | | 372 | |  | | 160 | |  | | | 159 | |  | |

Table S10. The effect of physical activity (PA) level on influenza antibody conversion, by strain and population (intervention). Predictors are by unit increase (Age and BMI) or versus dichotomous comparator (PA level and Sex). Estimates are given with 95%CI in parentheses. BMI = body mass index.

# Section III. Summary of findings

## Table S11. Effect of acute exercise on antibody response in PA and inactive persons

| Outcome (change in titer) | aOR (95%CI) | aOR (95%CI) | # participants (studies) | Quality of evidence* |
| --- | --- | --- | --- | --- |
| PA status: | PA | inactive |  |  |
| H1N1 | 1.14 (0.97-1.33) | 0.89 (0.70-1.10) | 527 (7) | Low |
| H3N2 | 0.97 (0.85-1.12) | 0.97 (0.78-1.18) | 540 (7) | Low |
| B | 0.95 (0.85-1.06) | 0.91 (0.77-1.07) | 541 (7) | Low |

## Table S12. Effect of being physically active on antibody response in persons undergoing acute exercise

| Outcome (change in titer) | aOR (95%CI) | aOR (95%CI) | # participants (studies) | Quality of evidence* |
| --- | --- | --- | --- | --- |
| Intervention status: | Acute exercise | Rested control |  |  |
| H1N1 titer change | 1.20 (1.03-1.40) | 0.95 (0.76-1.18) | 527 (7) | Low |
| H3N2 titer | 0.96 (0.84-1.10) | 0.94 (0.76-1.167) | 540 (7) | Low |
| B titer | 0.99 (0.89-1.11) | 1.01 (0.85-1.19) | 541 (7) | Low |

*Quality of evidence was informally judged based on GRADE handbook (https://gdt.gradepro.org/app/handbook/handbook.html#h.svwngs6pm0f2) criteria. GRADE is a useful tool however it increases quality of evidence where a large magnitude of effect is found. We believe null findings are meaningful and can be based on a range of quality of evidence; therefore, we used the criteria as a guide. We defined moderate quality as “further research would be likely to impact our confidence in the estimate of effect and may change the estimate” and low quality evidence as very likely to do so. In particular, we considered the following:

- Consistency/Directness: although the interaction effect was not possible to determine on a study-by-study basis, we note across studies the effect of acute exercise on titer change varied, was generally small and statistically weak with no suggestion of dose effect when considering the study proportion sufficiently active PA. That is, in studies comprised 43-91% sufficiently PA participants there was no evidence that proportion related to the effect of acute exercise.
- Differences across studies in PA measurement also downgraded the quality of evidence. Differences across studies in population age and intervention type were addressed via sub-analyses.
- Potential bias issues as raised by the risk of bias outcome.

# IV. References

1. Long JE, Ring C, Drayson M, Bosch J, Campbell JP, Bhabra J, et al. Vaccination response following aerobic exercise: Can a brisk walk enhance antibody response to pneumococcal and influenza vaccinations? Brain, Behavior, and Immunity. 2012;26(4):680-7. doi: 10.1016/j.bbi.2012.02.004.

2. Ranadive SM, Cook M, Kappus RM, Yan H, Lane AD, Woods JA, et al. Effect of acute aerobic exercise on vaccine efficacy in older adults. Medicine and Science in Sports and Exercise. 2014;46(3):455-61. doi: 10.1249/MSS.0b013e3182a75ff2.

3. Bohn-Goldbaum E, Pascoe A, Singh MF, Singh N, Kok J, Dwyer DE, et al. Acute exercise decreases vaccine reactions following influenza vaccination among older adults. Brain, Behavior, & Immunity - Health. 2020;1:100009. doi: <https://doi.org/10.1016/j.bbih.2019.100009>.

4. Campbell JP, Edwards KM, Ring C, Drayson MT, Bosch JA, Inskip A, et al. The effects of vaccine timing on the efficacy of an acute eccentric exercise intervention on the immune response to an influenza vaccine in young adults. Brain, Behavior, and Immunity. 2010;24(2):236-42. doi: 10.1016/j.bbi.2009.10.001.

5. Edwards KM, Burns VE, Allen LM, McPhee JS, Bosch JA, Carroll D, et al. Eccentric exercise as an adjuvant to influenza vaccination in humans. Brain, Behavior, and Immunity. 2007;21(2):209-17. doi: 10.1016/j.bbi.2006.04.158.

6. Edwards KM, Burns VE, Reynolds T, Carroll D, Drayson M, Ring C. Acute stress exposure prior to influenza vaccination enhances antibody response in women. Brain, Behavior, and Immunity. 2006;20(2):159-68. doi: 10.1016/j.bbi.2005.07.001.

7. Edwards KM, Campbell JP, Ring C, Drayson MT, Bosch JA, Downes C, et al. Exercise intensity does not influence the efficacy of eccentric exercise as a behavioural adjuvant to vaccination. Brain, Behavior, and Immunity. 2010;24(4):623-30. doi: 10.1016/j.bbi.2010.01.009.

8. Lee VY, Bohn-Goldbaum E, Fong J, Barr IG, Booy R, Edwards KM. Analgesic and adjuvant properties of exercise with vaccinations in healthy young population. Hum Vaccin Immunother. 2021:1-7. doi: 10.1080/21645515.2020.1859322.

9. Elzayat MT, Markofski MM, Simpson RJ, Laughlin M, LaVoy EC. No Effect of Acute Eccentric Resistance Exercise on Immune Responses to Influenza Vaccination in Older Adults: A Randomized Control Trial. Frontiers in Physiology. 2021;12(1279). doi: 10.3389/fphys.2021.713183.

10. Housel LA, Beltran TA, Spooner C, Collins LC, Jr., Ewing DF, Williams M, et al. A randomized controlled trial of NSAIDs or exercise to reduce delayed local pain after influenza vaccination. Journal of Allergy and Clinical Immunology: In Practice. 2021;9(2):1018-20.e1. doi: 10.1016/j.jaip.2020.08.058.

11. Colburn A, Wright S, Lopez V, Giersch G, Belval L, Hosokawa Y, et al. Aerobic exercise and environmental heat stress as adjuvants to seasonal influenza vaccine. FASEB Journal Conference: Experimental Biology. 2018;32(1 Supplement 1). doi:

12. Edwards KM, Pascoe AR, Fiatarone-Singh MA, Singh NA, Kok J, Booy R. A randomised controlled trial of resistance exercise prior to administration of influenza vaccination in older adults. Brain, Behavior, and Immunity. 2015;49 (Supplement 1):e24-e5. doi:
